# Elevated levels of Wnt signaling disrupt thymus morphogenesis and function

**DOI:** 10.1038/s41598-017-00842-0

**Published:** 2017-04-11

**Authors:** Jeremy B. Swann, Christiane Happe, Thomas Boehm

**Affiliations:** Department of Developmental Immunology, Max Planck Institute of Immunobiology and Epigenetics, Stuebeweg 51, D-79108 Freiburg, Germany

## Abstract

All vertebrates possess a thymus, whose epithelial microenvironment is essential for T cell development and maturation. Despite the importance of the thymus for cellular immune defense, many questions surrounding its morphogenesis remain unanswered. Here, we demonstrate that, in contrast to the situation in many other epithelial cell types, differentiation of thymic epithelial cells (TECs) proceeds normally in the absence of canonical Wnt signaling and the classical adhesion molecule E-cadherin. By contrast, TEC-intrinsic activation of β-catenin-dependent Wnt signaling blocks the morphogenesis of the thymus, and overexpression of a secreted Wnt ligand by TECs dominantly modifies the morphogenesis not only of the thymus, but also of the parathyroid and thyroid. These observations indicate that Wnt signaling activity in the thymus needs to be precisely controlled to support normal TEC differentiation, and suggest possible mechanisms underlying anatomical variations of the thymus, parathyroid and thyroid in humans.

## Introduction

The epithelial microenvironment of the thymus exhibits a unique cellular architecture, which strains the standard definitions of epithelial structures, both in anatomy and function. Anatomically, epithelial cells are typically arranged into stereotypical cell layers; functionally, they are usually categorized as having absorptive, secretory or barrier roles. By contrast, thymic epithelial cells (TECs) are arrayed in a complex, reticulated configuration, and seemingly lack adsorptive, secretory or barrier functions. Instead, the unusual TEC architecture is thought to support the functional role of the thymus - the recruitment of lymphoid progenitors, the induction of T cell development and the quality control of the emerging repertoire of somatically diversified antigen receptors for self-tolerance^1^. Despite defying standard classifications for epithelial cells, the epithelial nature of TECs is demonstrated by their expression of a range of keratins, and their embryological origin from the pharyngeal endoderm^2^.

The thymic epithelium arises from the third pharyngeal pouch (3PP). In mice, the 3PP forms at around embryonic day 10.5 (E10.5); it consists of a simple endodermal cell layer divided into two domains, each fated to become the epithelial component of either the thymus or the parathyroid. These domains can be identified by the expression of two key transcription factors, Gcm2 and Foxn1^3^. *Gcm2* begins to be expressed at ≈E10.5 in the epithelial cells located in the anterior-dorsal region of the pouch and specifies a parathyroid fate, while *Foxn1* is expressed slightly later at ≈E11.0 in the epithelial cells of the posterior-ventral region of the pouch, and specifies the adoption of a thymic fate. Exactly how these expression domains are established is not entirely clear, however, with respect to the thymic rudiment BMP^4–6^, Shh^7^ and Wnt signaling^8^ have all been implicated in the induction of *Foxn1* expression.

After patterning of the 3PP into parathyroid/thymus domains, the parathyroid and thymus anlagen separate and adopt organ-specific architectures and functions. The segregation of the parathyroid and thymic anlagen occurs at approximately the time when the nascent thymic lobes are first populated by lymphoid precursors, and the thymic epithelium transitions from a simple cell layer to a reticulated architecture. Simultaneously with the *en bloc* migration to their target destination in the mediastinum, the thymic lobes increase in size to accommodate a rapid expansion in thymocyte numbers, and become patterned into the stereotypical cortical and medullary domains.

While each of these events in thymus development have been extensively documented histologically, and several of the transcription factors^9–17^ and signaling molecules^4, 5, 18–25^ that regulate thymus and parathyroid specification and positioning have been identified, there is little information regarding the molecular underpinnings of many of the steps involved in the transition from 3PP to mature thymus.

E-cadherin (encoded by the *Cdh1* gene) is a classical cadherin involved in the formation of adherens junctions; its extracellular domain is composed of cadherin repeats that support homotypic interactions, its intracellular domain interacts with catenins in order to mediate connections with the cytoskeleton^26^. Previous studies demonstrated that E-cadherin is expressed by TECs^27^, and that blocking homotypic E-cadherin interactions prevents thymic lobe formation in re-aggregate thymus organ culture (RTOC) assays^28^, suggesting a functional role in thymus organogenesis. Intriguingly, β-catenin (encoded by the *Ctnnb1* gene), a critical binding partner of E-cadherin has also been implicated in thymus development^29^.

β-catenin is a multifunctional intracellular molecule involved in both cell-cell adhesion and Wnt signaling^30^. β-catenin modulates cell-cell adhesion by binding to the intracellular tail of the classical cadherins at cell membranes, mediating interactions with the actin filament network. In addition, β-catenin is a key component of the Wnt signaling pathway, where it serves as a transcriptional co-regulator to modulate the expression of Wnt target genes. Wnts play many roles in development, acting as morphogens or differentiation signals in a range of different organ systems^31, 32^. A number of studies have suggested that Wnt signaling and β-catenin play important roles in thymus development, architecture, function and senescence^29, 33–40^, although the relative contributions of β-catenin-dependent and -independent signaling are largely unexplored.

Given the proposed roles of E-cadherin and Wnt-signaling as modulators of thymus development, we examined – using a combination of conditional gene targeting and transgenic approaches – whether the interplay between cadherin-mediated cell-cell adhesion and Wnt signaling via β-catenin is responsible for guiding the migration of the thymic lobes, and/or for the establishment and maintenance of the specialized thymic epithelial architecture during embryogenesis.

## Results

### Characteristics of Wnt signaling in the thymus

**Figure 1:**
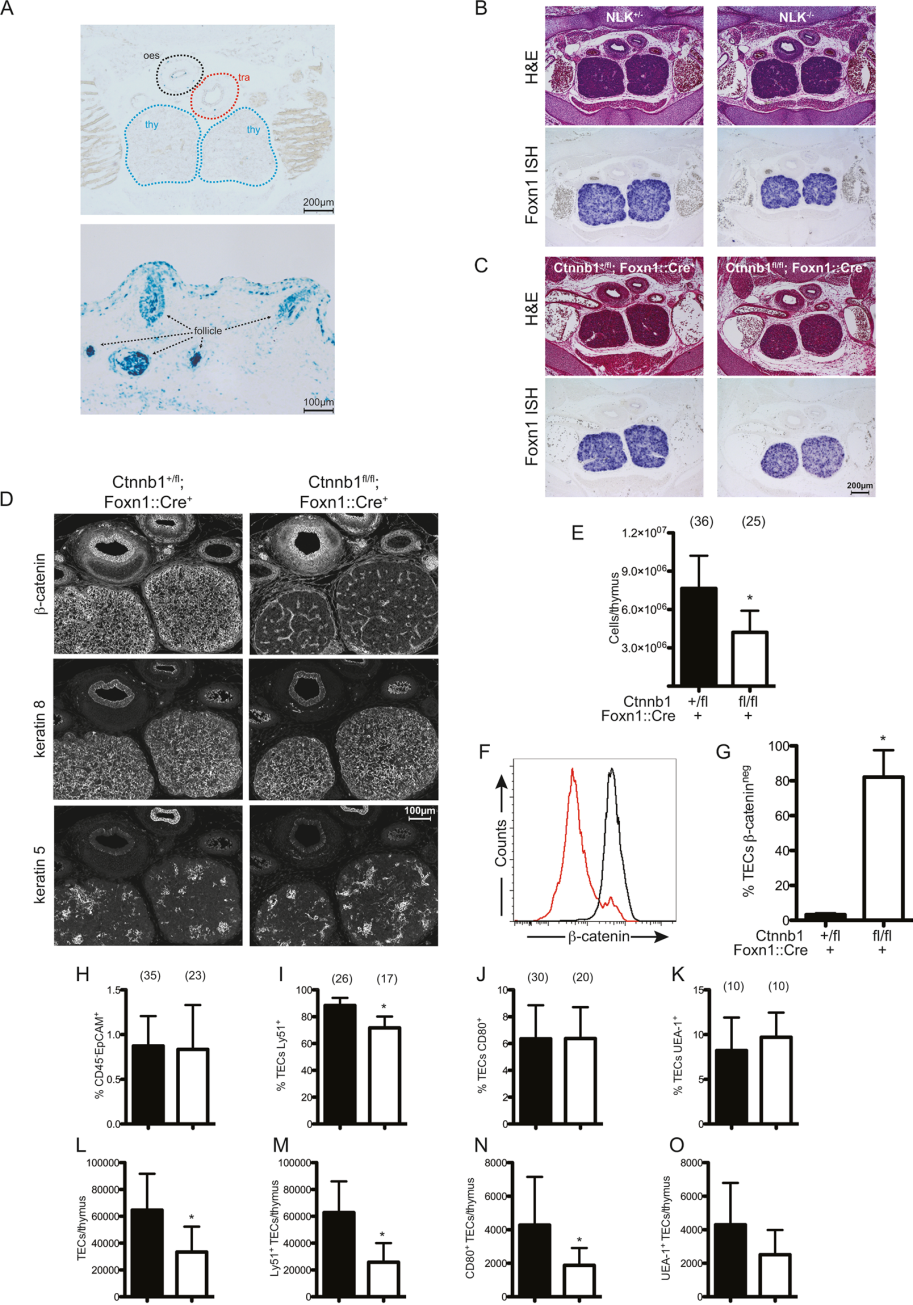
Thymus development in *Nlk*- and *Ctnnb1*-deficient mice. (**A**) X-Gal staining of E15.5 BATgal-transgenic thymus (upper panel) and whisker follicles (lower panel). (**B**,**C**) Thymus sections stained with H&E (top panels), or subjected to *Foxn1* RNA *in situ* hybridization (ISH) (bottom panels). (**D**) Immunofluorescence (IF) analysis of thymus sections with antibodies against β-catenin, K8 and K5 for the indicated genotypes. E15.5 embryos of the indicated genotypes are shown in (**B**–**D**). Scale bars are indicated. (**E**) Total thymocyte cell counts. (**F**) Intra-cellular staining for β-catenin expressed by gated CD45^−^EpCAM^+^ TECs (Control TECs, black, *Ctnnb1-*deficient TECs, red). (**G**) Percentage of β-catenin-negative TECs. (**H** and **L**) Proportions (**H**) and total numbers (**L**) of EpCAM^+^ TECs. (I&M) Proportions (**I**) and total numbers (**M**) of Ly51^+^ TECs. (J&N) Proportions (**J**) and total numbers (**N**) of CD80^+^ TECs. (K&O) Proportions (**K**) and total numbers (**O**) of UEA-1^+^ TECs. Analyses of TECs from newborn control (black columns) and *Ctnnb1-*deficient (open columns) mice are shown in (**E**–**O**). Bar graphs are presented as mean ± SD.

We confirmed the results of previous work indicating that thymic epithelial cells express several Wnts, including the so-called canonical Wnt10a and Wnt10b and the non-canonical Wnt4 ligands^41^. However, despite high expression levels of canonical Wnts in the thymus, canonical Wnt signaling to the nucleus appears to be low, when examined by a transgenic reporter (BATgal^42^); this outcome is in contrast to the situation in the Foxn1-expressing whisker follicle, where the activity of the reporter is high (Fig. 1A). This unexpected discrepancy prompted us to conduct a detailed examination of the physiological roles of β-catenin-dependent and β-catenin-independent Wnt signaling in the thymic epithelium. Our strategy was based on *in vivo* perturbation experiments via transgenic modulation of several pathway components; the outcomes of a total of eight different transgenically modified signaling environments (see Supplementary Figure 1 for a schematic representation) are reported here.

### NLK and β-catenin are not essential for the formation of the embryonic thymus

Wnt signaling occurs via β-catenin-dependent and β-catenin-independent pathways (Supplementary Figure 1A). NLK is a potent modulator of β-catenin-independent Wnt signaling^43–45^, whereas β-catenin is a central component of the classical pathway. Hence, we examined thymus development in two mouse lines with defects in either the β-catenin-independent signaling pathway (*Nlk*-deficient mice, Supplementary Figure 1B) or the β-catenin-dependent signaling pathway (conditional *Ctnnb1-*deficient mice, Supplementary Figure 1C). Histological examination of E15.5 *Nlk* ^+/−^ and *Nlk* ^−/−^ embryos revealed a structurally normal thymus in the upper mediastinum; importantly, expression of *Foxn1*, encoding the master regulator of thymic epithelial differentiation^46, 47^ was found to be normal (Fig. 1B). Collectively, these findings indicated that NLK-dependent Wnt signaling is not required for the early stages of thymus development. The thymic lobes in *Ctnnb1-*deficient E15.5 embryos were correctly positioned in the mediastinum, and also exhibited robust expression of *Foxn1* transcripts (Fig. 1C), suggesting that canonical Wnt signaling in the thymic epithelium is dispensable for at least early stages of thymopoiesis. This unexpectedly mild thymus phenotype prompted us to further characterize thymus development in *Ctnnb1-*deficient mice.

### β-catenin modulates thymic cellularity, but not TEC differentiation in the embryonic thymus

At E15.5, *Ctnnb1-*deficient thymi appeared histologically normal, as indicated by keratin-5 (K5) and keratin-8 (K8) staining (Fig. 1D), whereas thymi from newborn *Ctnnb1-*deficient mice revealed a ≈50% reduction in cellularity (Fig. 1E). The majority of TECs were β-catenin deficient (Fig. 1F and G); and their number was reduced by about 50% (Fig. 1H and L). Further phenotypic characterization of cortical TEC (cTEC) and medullary TEC (mTEC) subsets (representative flow cytometry gating depicted in Supplementary Figure 2) indicated that Ly51^+^ cTECs were slightly reduced in *Ctnnb1-*deficient mice (Fig. 1I and M), whereas proportions of CD80^+^ (Fig. 1J and N) and UEA-1^+^ mTECs (Fig. 1K and O) were unchanged. Although total thymus cellularity and TEC numbers were already significantly reduced in *Ctnnb1-*deficient mice by E15.5 (Supplementary Figure 3), the differentiation into cTEC and mTEC compartments is largely unperturbed, at least until the newborn stage. Mice rendered β-catenin-deficient using the *Foxn1::Cre* system used here die within a few days of birth due to defects in skin development, where *Foxn1* is also expressed in the suprabasal layer^48^. These findings support the notion that thymic epithelial cells and skin keratinocytes exhibit distinct architectures of Wnt signaling as revealed by the BATgal reporter (Fig. 1A); however, this early lethality precluded the analysis of later stages of thymus development.

### TEC-intrinsic over-expression of β-catenin perturbs thymus migration and differentiation

**Figure 2:**
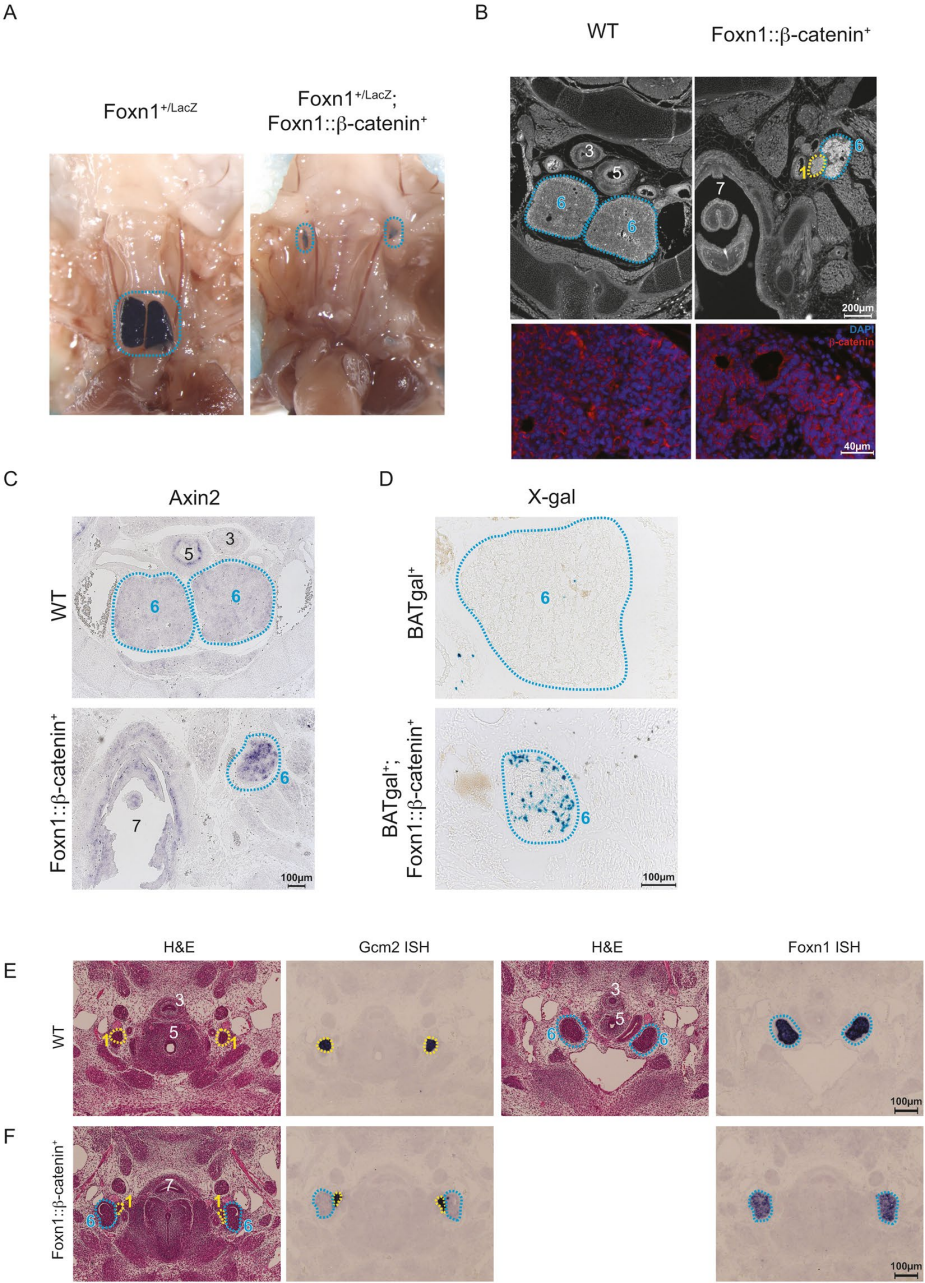
Defective thymus development due to TEC-intrinsic β-catenin overexpression. (**A**) Ectopic location of the thymic lobes in Foxn1::β-catenin-transgenic mice. Foxn1-expressing thymic lobes are labelled blue by wholemount X-Gal staining. (**B**) Ectopic location of thymic lobes and parathyroid in E15.5 β-catenin-transgenic embryos, visualised by staining for β-catenin (upper panels 20x panoramic view, lower panels thymus tissue at 40x magnification with DAPI counterstain). (**C**) RNA *in situ* hybridization for *Axin2* on thymus sections of E15.5 embryos. (**D**) Activity of the BATgal reporter allele in the thymus of E15.5 embryos. (**E**) Development of the parathyroid and thymus of wild-type mice at E12.5 examined by H&E staining and RNA *in situ* hybridization with the indicated gene-specific probes. (**F**) Development of the parathyroid and thymus of mutant mice at E12.5 examined as in (**E**). A numerical key for the histological labels can be found in Fig. 3.

Due to the dual functions of β-catenin, the reduced numbers of hematopoietic cells in the thymus of *Ctnnb1-*deficient mice could be the (indirect) result of either defects in epithelial Wnt signaling pathway activity, or due to altered cell-cell adhesion via classical cadherins. To investigate if activation of canonical Wnt signaling in TECs can perturb their development, we expressed a stabilized form of β-catenin in TECs (Supplementary Figure 1D). No thymus tissue could be detected in the thoracic cavity of newborn *Foxn1::β-catenin*-transgenic mice additionally transgenic for a *Foxn1* ^lacZ^-reporter allele^47^; instead, small, ectopic thymic lobes were found in the cervical region, approximately level with the thyroid cartilage (Fig. 2A). In E15.5 embryos, ectopic thymic tissue is found arrested laterally to the carotid artery, approximately at the level of the cricoid cartilage. The characteristic epithelial cell network observed in normal thymi had collapsed into a cystic structure composed of tangled squamous epithelium (Fig. 2B). Activation of the Wnt signaling pathway in TECs of *Foxn1::β-catenin* transgenic mice was confirmed by increased expression levels of *Axin2* (Fig. 2C), a known β-catenin target gene, and β-galactosidase expression in *BATgal* reporter mice^42^ (Fig. 2D). Collectively, these results indicate that activated Wnt-signaling in TECs perturbs thymus migration and TEC differentiation, and hence demonstrates that under physiological conditions β-catenin-dependent Wnt signaling is rendered inactive in the thymic epithelium.

**Figure 3:**
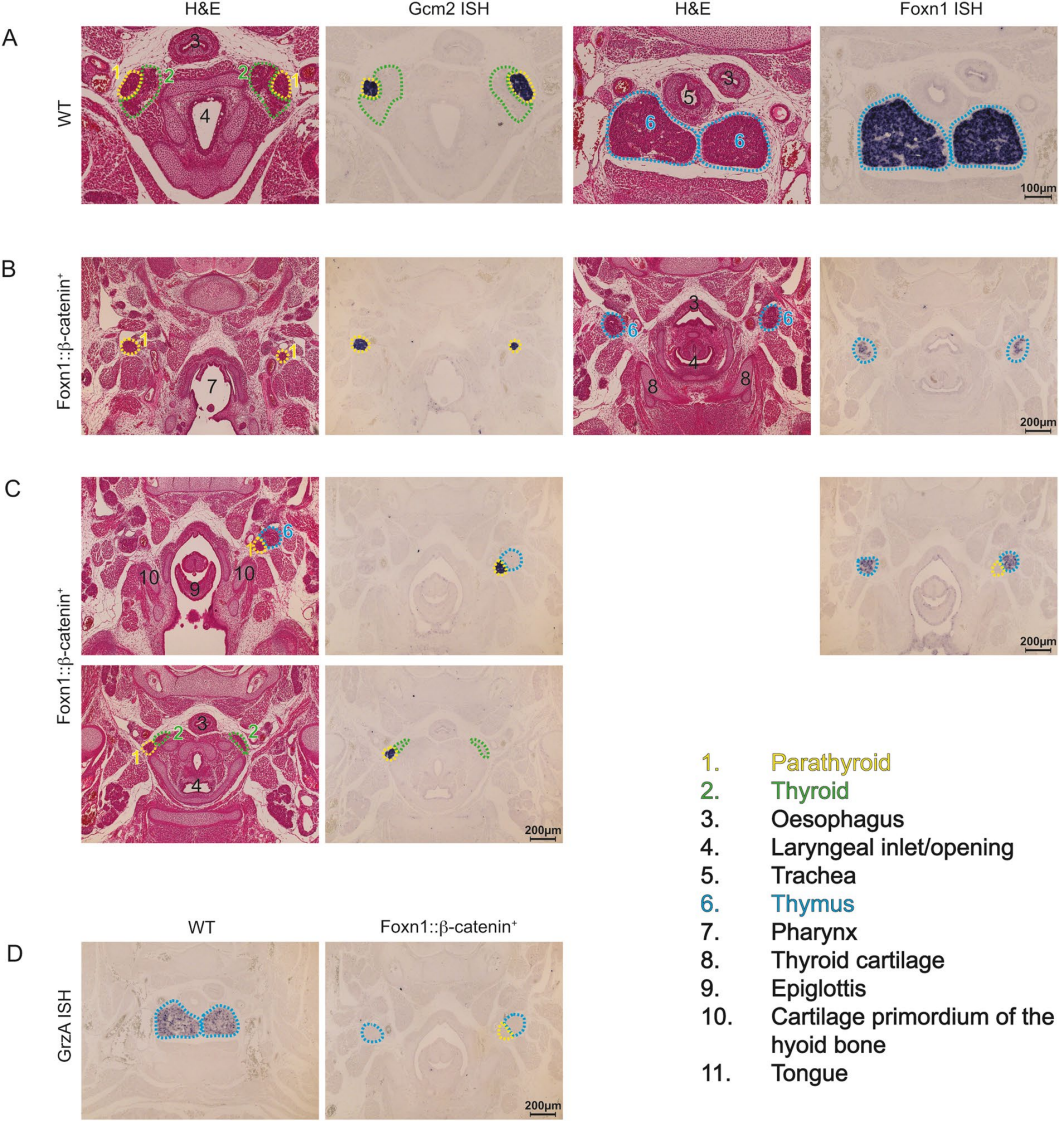
TEC-intrinsic β-catenin overexpression disrupts thymus and parathyroid development. (**A**) Development of the parathyroid and thymus of wild-type mice at E15.5 examined by H&E staining and RNA *in situ* hybridization with the indicated gene-specific probes. (**B**) Development of the parathyroid and thymus of mutant mice at E15.5 examined as in (**A**). (**C**) Development of the parathyroid and thymus of mutant mice at E15.5 examined as in (**A**); sections are derived from the same embryo, with the top row depicting the ectopic location of the left parathyroid lobe and the thymic lobes (approximately at the level of the pharyngeal inlet), and the second row demonstrating the position of the right parathyroid in the expected position at the anterior pole of the thyroid. The numerical figure label key applies to all figures. In (**A**–**C**), H&E stained sections are matched with corresponding sections subjected to RNA *in situ* hybridization. (**D**) RNA *in situ* hybridization with a *GrzA* probe to detect developing thymocytes.

In contrast to wild-type controls (Fig. 2E), the thymus and parathyroid of *β-catenin*-transgenic mice were ectopically located at E12.5, and had failed to separate from one another (Fig. 2F). These defects were even more prominent by E15.5. In wild-type E15.5 embryos, the parathyroid lobes have completed their migration to the anterior poles of the thyroid, and the thymic lobes are positioned together in the upper mediastinum (Fig. 3A). The thymic lobes in *β-catenin*-transgenic embryos failed to migrate and were instead located at the level of the bifurcation of the esophagus and larynx in the form of a malformed rudiment (Fig. 3B and C). The position of the parathyroid lobes in *β-catenin*-transgenic mice was variable; they were most commonly found adjacent to the defective thymic rudiment, often showing incomplete segregation (Fig. 3B, and upper panel in Fig. 3C), although occasionally an individual parathyroid lobe appeared to have migrated to the very anterior pole of the thyroid (Fig. 3C, lower panel). Despite their ectopic location, the parathyroid lobes were seemingly functional, as *β-catenin*-transgenic mice survived to adulthood without overt evidence of hypo-parathyroidism. The rudimentary thymus present in *β-catenin*-transgenic mice expressed lower levels of *Foxn1*-transcripts than wild-type controls at E12.5 (Fig. 2E and F) and E15.5 (Fig. 3A compared to 3B and C). Expression of a *Foxn1::EGFP* reporter was similarly reduced in three-day-old *β-catenin*-transgenic mice (Supplementary Figure 4A); as a consequence of reduced *Foxn1* expression, the rudimentary thymic lobes of *β-catenin*-transgenic embryos failed to be colonized by Granzyme A-expressing hematopoietic cells^49^ (Fig. 3D). This failure of T-lymphopoiesis persisted throughout development, resulting in severe T cell hypoplasia in adult *β-catenin-*transgenic mice (Supplementary Figure 4B). Overall, this data demonstrates that overexpression of stabilized β-catenin causes defective migration of the thymic anlagen, and reduces *Foxn1* expression, thus preventing the formation of the physiological reticulated TEC architecture. Interestingly, TEC-specific over-expression of stabilized β-catenin was also sufficient to perturb the migration, but not the function, of the parathyroids.

### E-cadherin is not required for normal thymus development

**Figure 4:**
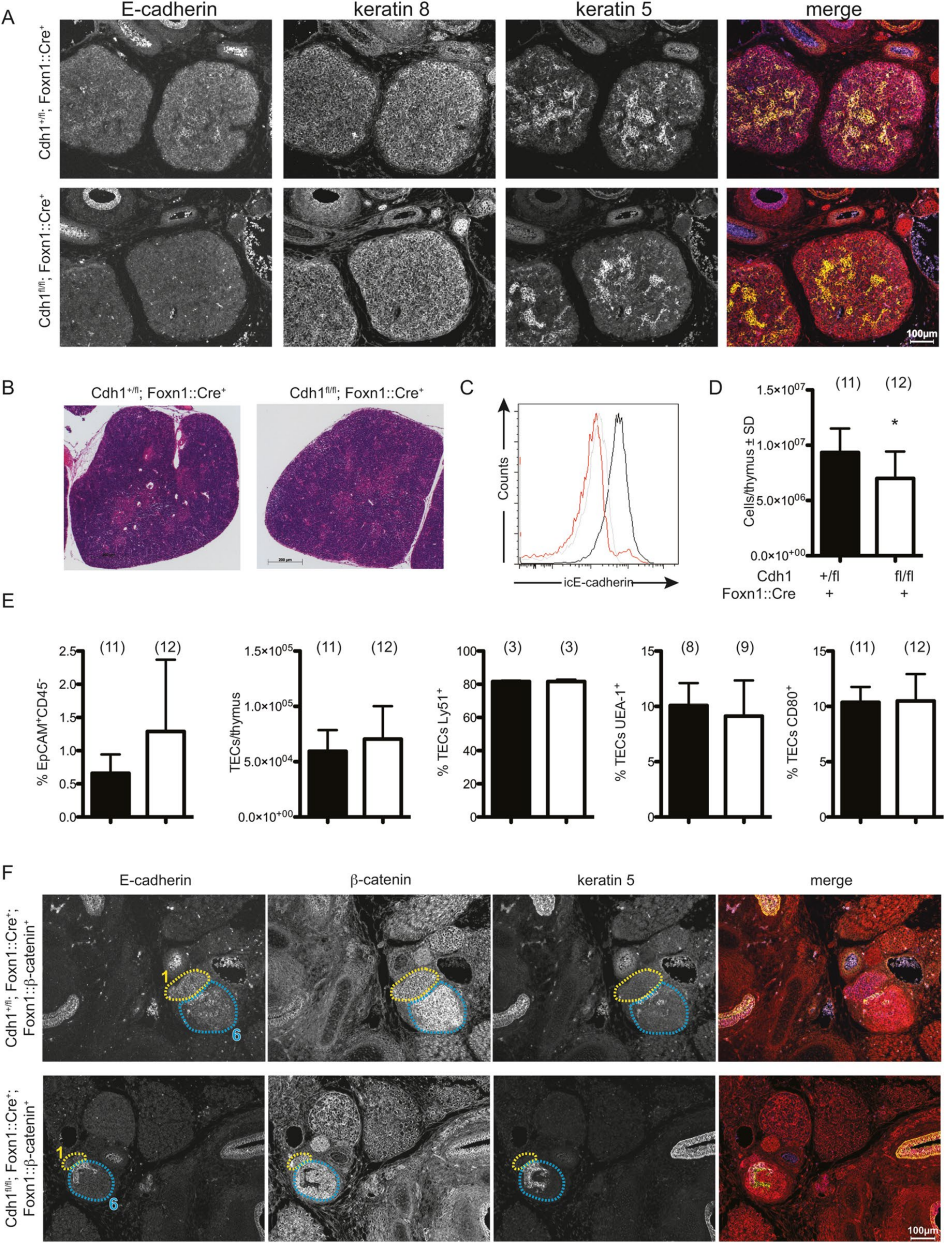
E-cadherin is not required for normal thymus development. (**A**) Immunofluorescence analyses of thymus sections of E15.5 control (top row) or E-cadherin deficient (bottom row) embryos stained with antibodies against the indicated antigens. (**B**–**E**) Analysis of thymi from newborn control and E-cadherin-deficient mice. (**B**) H&E staining. (**C**) Staining for intracellular E-cadherin in CD45^−^EpCAM^+^ TECs from control (black tracing) or E-cadherin deficient (red tracing) mice; isotype control staining (grey tracing). (**D**) Total cell numbers in the thymus. (**E**) Proportion and absolute numbers of total TECs, proportions of Ly51^+^, UEA-1^+^ and CD80^+^ TECs in control (black bars) and E-cadherin-deficient (open bars) thymi. In D and E, data are depicted as mean ± SD. (**F**) Immunohistochemistry analyses of E15.5 E-cadherin-sufficient or -deficient Foxn1::β-catenin-transgenic embryos with antibodies against the indicated antigens.

Given the dual roles of β-catenin in both Wnt signaling and cell adhesion, we determined if the defects observed in *Ctnnb1-*mutant mice could be recapitulated by ablation of E-cadherin (Supplementary Figure 1E). However, the defects observed in *in vitro* studies^28^ were not seen with our *in vivo* model. E-cadherin-deficient thymi were correctly positioned within the mediastinum, with normal patterning of the thymic lobes into cortical and medullary zones (Fig. 4A and B). In newborn mice, the deletion of E-cadherin (Fig. 4C) was accompanied by a slight decrease in total thymic cellularity (Fig. 4D), whereas total numbers of TECs, and the proportions of Ly51^+^, UEA-1^+^ and CD80^+^ TECs were equivalent to littermate controls (Fig. 4E). No defects were noted in adult mice lacking E-cadherin in TECs (Supplementary Figure 5). Collectively, these results demonstrated that E-cadherin is dispensable for thymus development *in vivo*.

### Defective migration of β-catenin-overexpressing thymi is not mediated via E-cadherin

Next, we examined a possible role of E-cadherin in the abortive thymus migration observed in *β-catenin*-transgenic mice, as increased levels of β-catenin have previously been noted to alter cellular adhesiveness via cadherins^50^. Ablation of E-cadherin expression in *β-catenin*-overexpressing TECs (Supplementary Figure 1F) did not however modify the phenotype. In E15.5 *Cdh1* ^*fl/fl*^ *; Foxn1::Cre; Foxn1::β-catenin* embryos, the thymus remained ectopic, and was unable to support T cell development (Fig. 4F), Whereas dosage of E-cadherin modified the *β-catenin*-overexpression phenotype in the skin of adult mice (Supplementary Figure 6A), thymopoietic function did not follow this pattern (Fig S6B and C). We conclude that the ectopic location of the thymic lobes and the loss of reticulated TEC architecture is a consequence of activated Wnt signaling in *β-catenin*-transgenic embryos is not caused by altered E-cadherin-mediated cell-cell interactions between TECs.

### Wnt4 overexpression disrupts thymus migration and TEC differentiation

**Figure 5:**
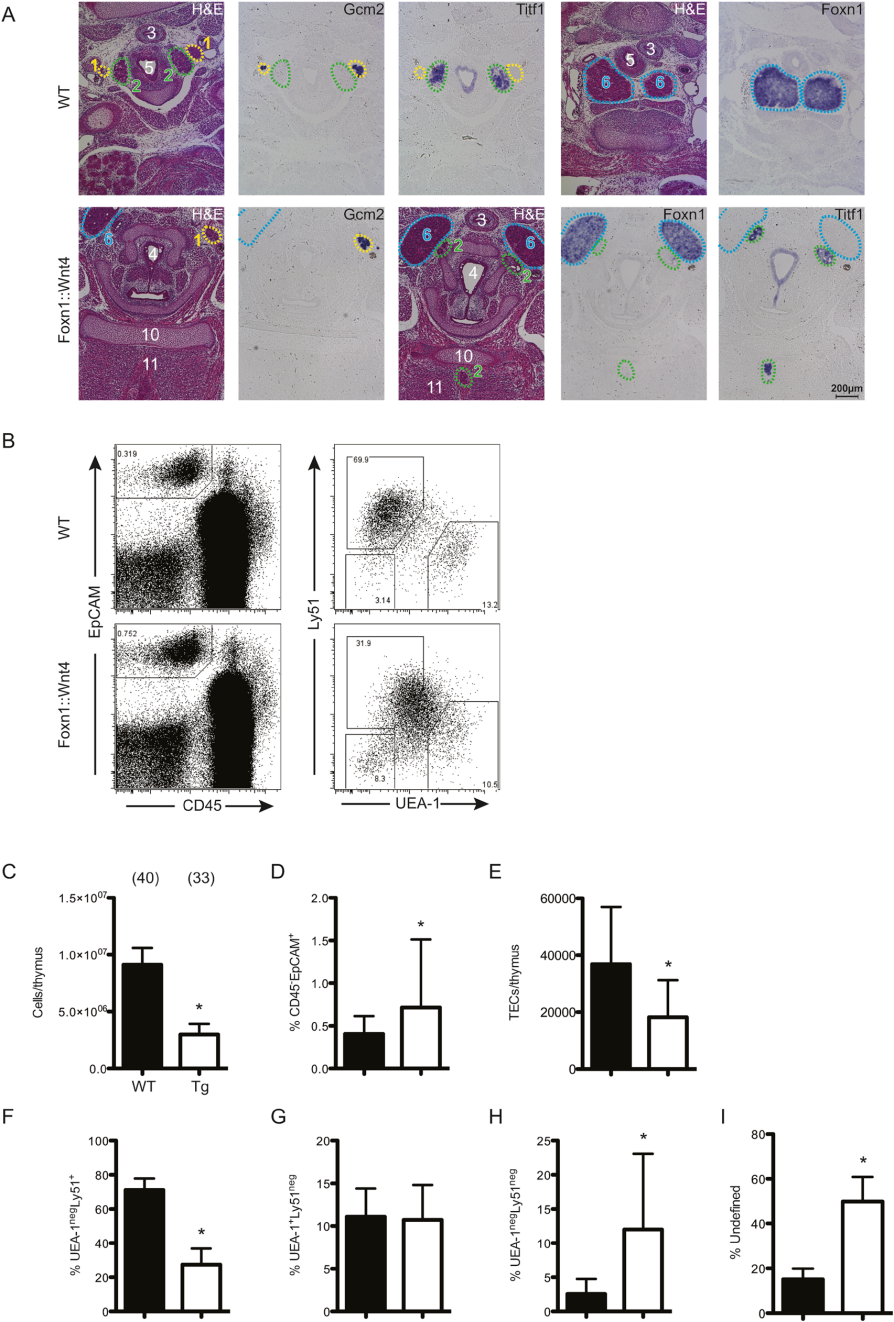
Wnt4-overexpression disrupts thymus migration and TEC differentiation. (**A**) Development of wild-type mice at E15.5 examined by H&E staining and RNA *in situ* hybridization with the indicated gene-specific probes (*Gcm2* [parathyroid], *Titf1* [thyroid] and *Foxn1* [thymus]). (**B**–**I**) Flow cytometric analysis of TECs from newborn wild-type and *Foxn1::Wnt4-*transgenic mice. (**B**) Representative gating of EpCAM^+^ TECs. Gates defining UEA-1^neg^Ly51^+^, UEA-1^+^Ly51^−^ and UEA-1^neg^Ly51^neg^ populations are depicted; events falling outside of these gates are classified as “undefined”. (**C**–**I**) Cell numbers and proportions of various TEC subsets (wild-type, black bars; open bars, *Foxn1::Wnt4*-transgenic mice). Means ± SD.

Wnt4 is able to trigger both β-catenin-dependent and β-catenin-independent signaling in a context-dependent manner, and has previously been demonstrated to play a role in embryonic thymus development^33^. Transgenic overexpression of *Wnt4* in TECs (Supplementary Figure 1G) resulted in ectopic thymus development, at E15.5 the thymic lobes were located adjacent to the trachea, approximately at the level of the thyroid cartilage (Fig. 5A). Interestingly, *Wnt4*-transgenic mice also exhibited ectopic parathyroid and thyroid tissue. The parathyroid anlagen were positioned asymmetrically, typically adjacent to the thymic anlagen, instead of being associated with the thyroid (Fig. 5A). Morphogenesis of the thyroid was also disrupted; some *Wnt4-*transgenic embryos exhibited additional ectopic thyroid tissue, variably located ventral to the trachea, or embedded in the septum of the developing tongue (Fig. 5A). In contrast to those in *β-catenin*-transgenic embryos, the ectopic thymic lobes of *Wnt4*-transgenic embryos were larger in size, exhibited a reticulated TEC architecture, and were colonized by developing thymocytes. In newborn wild-type mice, three TEC populations can be distinguished on the basis of UEA-1 and Ly51 expression: UEA-1^+^Ly51^−^ mTECs, Ly51^+^UEA-1^−^ cTECs and a UEA-1^−^Ly51^−^ double negative population (Fig. 5B). The cellularity of *Wnt4*-transgenic thymi (Fig. 5C), including TECs (Fig. 5D and E) was substantially decreased. UEA-1 and Ly51 staining revealed a substantial alteration in TEC subpopulations in *Wnt4*-transgenic mice (Fig. 5B). Although Ly51^+^ and UEA-1^+^ TECs could be detected in transgenic mice, we observed a significant increase in the proportions of UEA-1^−^Ly51^−^ TECs, and a dramatic increase in an undefined population of TECs with intermediate staining levels for both UEA-1 and Ly51 (Fig. 5F to I). Altered TEC development persists into adulthood (Supplementary Figure 7A); 12 week-old *Wnt4*-transgenic mice exhibited decreased proportions of UEA-1^+^ TECs and increased proportions of Ly51^+^ and UEA-1^−^Ly51^−^ TECs, together with increased proportions of undefined TECs expressing variable levels of UEA-1 and Ly51 (Supplementary Figure 7B), indicating Wnt4-overexpression either disrupts TEC differentiation, or selectively promotes the survival of atypical TEC subsets. At this age, the numbers of thymocytes were still reduced compared to controls, although the proportions of the various thymocyte subsets were normal (Supplementary Figure 7C).

### Defective migration of Wnt4-transgenic thymi is independent of TEC-intrinsic β-catenin and NLK signaling

**Figure 6:**
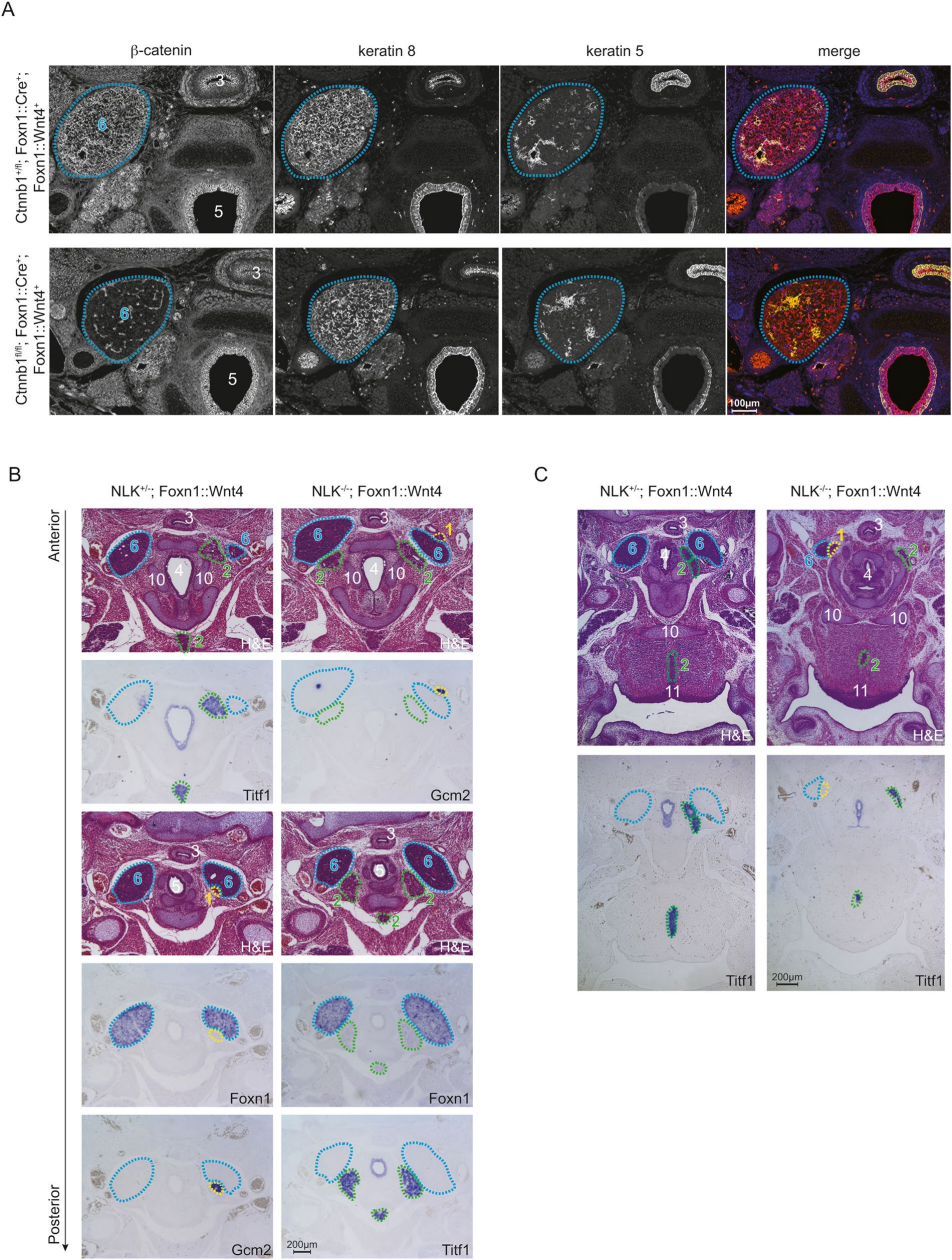
Deficiency in β-catenin or Nlk fails to correct defective organ migration induced by Wnt4-overexpression. (**A**) Immunohistochemistry analysis of E15.5 β-catenin-sufficient (top row) and *Ctnnb1-*deficient (bottom row) *Foxn1::Wnt4*-transgenic embryos. (**B**) Development of thyroid, thymus and parathyroid in E15.5 *Nlk*-sufficient (left panels) and *Nlk-*deficient (right panels) *Foxn1::Wnt4*-transgenic embryos analyzed as in Fig. 5A. (**C**) Ectopic thyroid tissue in the septum of the tongue identified by H&E staining and RNA *in situ* hybridization with a *Titf1* probe. Ectopic thyroid tissue located ventral to the trachea can also be observed in (**B**), second to top panel in the left column, bottom panel in the right column.

Given that Wnt4 can activate both β-catenin-dependent and β-catenin-independent signaling, we next sought to determine if the defective migration of *Wnt4*-overexpressing thymi could be modified by simultaneous ablation of either β-catenin or NLK. Deficiency of *Ctnnb1* in *Wnt4*-transgenic embryos (*Ctnnb1* ^*fl/fl*^ *; Foxn1::Cre; Foxn1::Wnt4*, Supplementary Figure 1H) did not alter the defective migration of *Wnt4-*transgenic thymi (Fig. 6A). Hence, the ectopic position of the thymic lobes in *Wnt4-*transgenic mice must be the result of either β-catenin-independent autocrine Wnt signaling in TECs, and/or secreted Wnt4 acting on non-TEC cell types. The latter possibility is indicated by the detrimental effects of the *Foxn1::Wnt4* transgene on parathyroid and thyroid development. NLK has previously been demonstrated to either enhance or suppress Wnt signaling in a context-dependent manner; however, the defective migration of the thymic lobes, parathyroids (Fig. 6B) or thyroid (Fig. 6B and C) was preserved in *Nlk-*deficient, *Wnt4-*transgenic embryos (Supplementary Figure 1I), indicating that NLK is not required to mediate the Wnt4-induced signaling pathway that disrupts organ morphogenesis.

### Combining Wnt activation and BMP inhibition exacerbates defective organ migration

**Figure 7:**
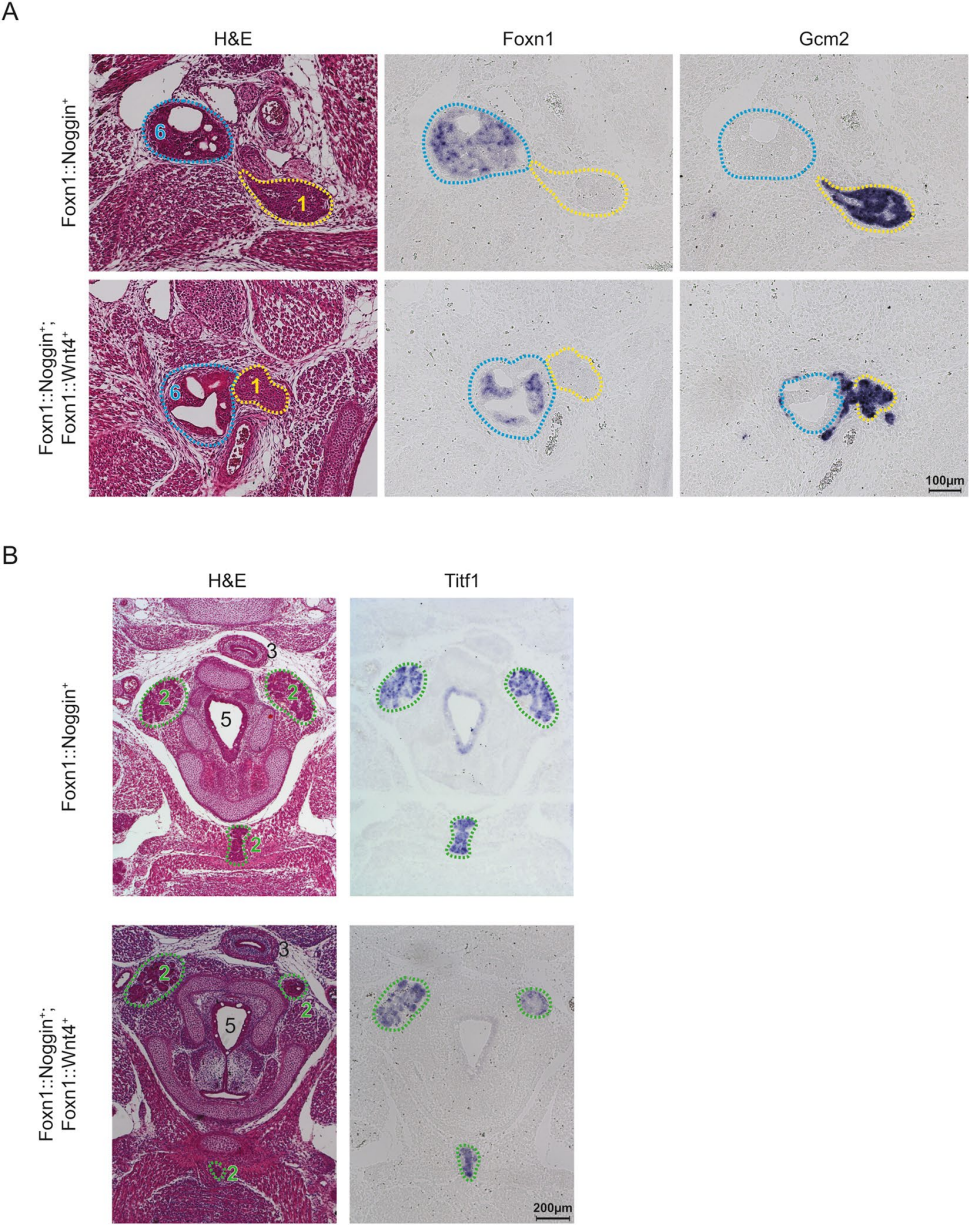
Combining BMP-inhibition with Wnt4-overexpression exaggerates organ migration defects. (**A**) Development of thymus and parathyroid in E15.5 *Foxn1::Noggin*-transgenic (top rows) or *Foxn1::Noggin;Foxn1::Wnt4-*double transgenic embryos (bottom rows) analysed as in Fig. 5A. (**B**) Development of the thyroid in E15.5 *Foxn1::Noggin*-transgenic (top rows) or *Foxn1::Noggin;Foxn1::Wnt4-*double transgenic embryos (bottom rows) as in Fig. 5A.

Inhibition of the BMP signaling pathway through the ectopic expression of the BMP-agonist Noggin results in ectopic thymus development^4^, a phenotype similar to that seen in mice with activated Wnt signaling. We therefore generated *Foxn1::Wnt4; Foxn1::Noggin* double-transgenic embryos to test if the defective thymus migration observed in *Wnt4-*transgenic mice could be enhanced or suppressed by inhibition of BMP signaling. The small thymic lobes of *Noggin*-transgenic embryos were ectopically positioned at the level of the hyoid cartilage (Fig. 7A) as previously reported, and were additionally shown to be associated with ectopic parathyroid tissue, located adjacent to the ectopic thymic lobes (Fig. 7A). In *Noggin;Wnt4*-double-transgenic embryos, we found that the thymic lobes were similarly positioned to those in single transgenic embryos, were further reduced in size, and exhibited a high degree of cystic transformation. The parathyroid cells in double-transgenic mice failed to properly segregate from the thymic anlage, with *Gcm2*-expressing cells found both surrounding the thymic lobe as scattered cells, or clustered in a partially disorganized lobe still attached to the thymus (Fig. 7A). *Noggin-* and *Wnt4-*single-transgenic embryos were also similar in the occurrence of ectopic thyroid tissue positioned ventrally from the thyroid cartilage (Fig. 7B). Ectopic thyroid tissue was present in 1/3 of *Noggin-*transgenic, 1/3 *Wnt4-*transgenic and 3/3 double-transgenic embryos. Collectively this data demonstrates that blocking BMP signaling, or activating Wnt signaling in the thymus can both modify not only thymus migration and development, but also disrupt normal development of the parathyroid and the thyroid; and that such defects can be exaggerated when BMP-inhibition and Wnt activation are combined.

## Discussion

The Wnt signaling pathway with its multitude of ligands, receptors and multi-functional intracellular mediators^51^ has a profound impact on development of many organs. However, the extent to which Wnt signaling impacts the development and function of the thymus has remained unclear. Here, using several epithelial cell-specific loss- and gain-of-function models, we demonstrate that thymus development can only proceed normally when β-catenin-dependent Wnt signaling is low or absent, and the extent of β-catenin-independent signaling does not exceed a certain threshold.

Previous *in vitro* studies demonstrated that treatment of thymic epithelium-derived cell lines with Wnts, or LiCl could induce the expression of *Foxn1*, implying that β-catenin-dependent Wnt signaling may be important for the expression of *Foxn1* during thymus development^8^. *In vivo*, however, we found that *Foxn1*-expression was readily detectable in *Ctnnb1-*deficient thymi. In addition, rather that enhancing *Foxn1* expression, we detected a decrease in *Foxn1* transcripts in TECs which overexpress stabilized β-catenin, consistent with a previous report^29^. Hence, although β-catenin-dependent Wnt signaling may fine-tune *Foxn1* expression levels, β-catenin is dispensable for *Foxn1* expression in the fetal thymic epithelium.

A second unexpected finding of our study was that E-cadherin is dispensable for thymus development. Previous work using *in vitro* models suggested that E-cadherin has an essential role in thymus organogenesis^28^, and that E-cadherin may have an important function in the human thymus as a mediator of epithelial-thymocyte crosstalk^52^. However, contrary to these reports we observed normal thymus development in mice in which E-cadherin was conditionally ablated in the thymic epithelium. In the skin, E-cadherin-deficiency is partially compensated for by the upregulation of P-cadherin^53^. Given the normal thymic architecture in *Ctnnb1-*deficient thymi observed here, it seems unlikely that such a mechanism is operative in the thymic epithelium, as the classical cadherins all utilize β-catenin to mediate their adhesive functions, and β-catenin was dispensable for the establishment of normal TEC architecture. Thus, our results rule out an essential role for E-cadherin in the formation of the unique reticulated epithelial network of TECs, and suggest that other types of adhesive interactions, for example tight-junctions formed by desmosomal cadherins, may underpin the stromal scaffold of the thymus. Alternatively, it remains possible that TEC architecture is preserved in β-catenin-deficient TECs by the recruitment of plakoglobin, a β-catenin paralog, to adhesive junctions^54^.

A striking outcome of this study was the finding that activation of Wnt signaling modulates the migration of not only the thymus, but also the parathyroid and thyroid. When Wnt signaling was activated in a TEC-intrinsic manner, organ morphogenic defects were restricted to the thymus and parathyroid, indicating that disruption of short-range cellular interactions within the common anlage of these two organs is sufficient to cause ectopic development. Moreover, paracrine signals emanating from the thymus have the potential to modulate the development of nearby organs, since morphogenic defects were observed not only for the thymus and parathyroid, but also the thyroid when the secreted ligand Wnt4 was overexpressed by TECs. Interestingly, ectopic thymus and thyroid tissue is occasionally observed in humans, and while typically benign, such ectopic tissue can in some instances be detrimental^55, 56^. Cervical thymus tissue has been detected in wild type mice^57^, with the frequency, position and number of ectopic thymic lobes differing among different strains, as well as between individuals within a given strain^57, 58^. These observations suggest that genetic background may influence the incidence of ectopic thymic tissue (inter-strain variation), while stochastic events may modify the final outcome (inter-individual variation). Additionally, although cervical thymic tissue is considered an anomaly in mice and humans, it is worth noting that the position and number of thymi lobes shows considerable inter-species variation, and that cervical thymic tissue is a normal feature of various vertebrate species, such as chickens^59^, sheep^60^ and wallabies^61^. We speculate that species-, strain- and individual-specific eccentricities in Wnt signaling pathways might account in part for the relatively broad phenotypic range of both normal and anomalous thymus anatomy.

Another unusual aspect of the Wnt4- and Noggin-overexpression models developed here was the detection of ectopic thyroid tissue either ventral to the trachea, or within the septum of the tongue. The presence of analogous ectopic thyroid tissue has been described in humans; a pyramidal lobe of the thyroid is a common variation of human thyroid anatomy, estimated to occur in approximately 40% of individuals^62, 63^, and the ectopic thyroid tissue observed in our study bears a striking resemblance to the pyramidal lobe tissue observed in human embryos^64^. Additionally, lingual thyroid^65^, is a known congenital anomaly present in up to 10% of the population^66^. The detection of pyramidal lobe-like and lingual thyroid tissue in *Wnt4*- and *Noggin*-transgenic mice might suggest that similar congenital abnormalities in humans, sometimes presenting as combined thyroid dysgenesis or agenesis with ectopic thymus tissue^67–69^ arise due to aberrations in Wnt or BMP signaling pathways during development.

In addition to being ectopically located, the thymus in *Wnt4*-transgenic mice demonstrated reduced cellularity. By contrast, when Wnt4 is overexpressed in hematopoietic progenitors transplanted into adult mice – rather than in the embryonic thymic stroma as is the case in our model - an increase in thymic cellularity is observed, associated with an expansion of lymphoid precursors in the bone marrow^37, 70^. Interestingly, in hematopoietic cells^71^, Wnt4 exerts its effect in a β-catenin-independent manner. This may also be true for TECs, since β-catenin-deficiency could not rescue the ectopic location of *Wnt4*-transgenic thymi, a result corroborated by the observation that the BATgal reporter, which is induced by β-catenin-dependent Wnt signaling^42^ displayed little activity in thymus. Collectively, this data indicates that Wnt signaling in the thymus predominantly occurs via β-catenin-independent pathway(s)^72^.

How might Wnt signaling modulate the development and migration of the pharyngeal apparatus? Perturbations in Wnt signaling in mesenchymal cells can result in ectopic thymus and parathyroid development^73^, demonstrating that normal development requires the appropriate level of Wnt signaling acting on a number of different target cell types. Several other signaling pathways such as BMP^4^, FGF^74^, ephrin^20^ and retinoic acid (RA)^21–23^ pathways have all been shown to modify thymus morphogenesis, raising the possibility that interaction(s) with these pathways may underpin the disturbances caused by activated Wnt signaling. Our observation that blocking BMP signaling exacerbated the morphogenic defects caused by *Wnt4*-overexpression clearly demonstrates that these pathways can interact to modify migration of pharyngeal endoderm-derived organs.

In conclusion, our results indicate that the classical cadherin E-cadherin is not required for the formation of the unique epithelial reticulum of the thymus. It also appears that the extent of β-catenin-dependent and -independent Wnt signaling in TECs must be precisely controlled to ensure normal development. Moreover, the activation of Wnt and inhibition of BMP signaling act as dominant modifiers of organ migration during morphogenesis of the thymus and other pharyngeal derivatives, indicating that these two signaling pathways have antagonistic roles.

## Methods

### Mice

Nlk-deficient^75^, Ctnnb1^fl^ ^76^, Cdh1^fl^ ^77^, Foxn1::Cre^78^, Foxn1::EGFP^57^, Foxn1::Noggin^4^ and BATgal^42^ mice have been previously described. The *Foxn1::*β*-catenin* transgene was constructed by cloning a Myc-tagged, stabilised form of β-catenin derived from plasmid pCS2MMBCS33A 6*Myc (a kind gift from Rolf Kemler) under the control of the *Foxn1* promoter. The stabilised β-catenin contains four serine-to-alanine phosphorylation site mutations (S33A/S37A/S41A/S45A) and a 6*Myc-tag at the C-terminus. The Myc-tagged, stabilised β-catenin cDNA was cloned into the NotI site of vector pAHB14, downstream of a 27,970 bp Foxn1 promoter fragment (corresponding to nucleotides 5680–33650 of Y12488)^4^. The *Foxn1::Wnt4* transgene was constructed by cloning a *Wnt4* cDNA fragment corresponding to nucleotides 43–1101 of NM_009523 into the NotI site of pAHB14. The *Wnt4* cDNA was amplified from newborn thymus cDNA with the primers Wnt4_f (5′ GAGGCGGCCGCACCATGAGCCCCCGTTCGTGCCT 3′) and Wnt4_r (5′ GAGGCGGCCGCTCACCGGCACGTGTGCATCTCC 3′), which introduce NotI restriction sites (underlined in the primer sequence). Transgene constructs were injected into FVB/N pronuclei using standard techniques, and the resulting transgenic mice were subsequently backcrossed to a C57BL/6J background.

For timed matings, the day of plug detection was designated as E0.5. All animal experiments were performed in accordance with relevant guidelines and regulations, approved by the review committee of the Max Planck Institute of Immunobiology and Epigenetics and the Regierungspräsidium Freiburg, Germany (license Az 35-9185.81/G-12/85).

### Histology

Embryos for H&E staining and ISH were fixed in 4% PFA and subsequently embedded in paraffin using standard techniques. Embryos used for X-Gal staining were fixed in 4% PFA for 1 hr on ice, infiltrated with 16% sucrose overnight, then frozen in OCT for sectioning. All embryo sections were cut in the transverse plane, in all figures dorsal is up, ventral is down. Scale bars are indicated.

### RNA *in situ* hybridization

RNA *in situ* hybridisation (ISH) on paraffin sections was performed using DIG-labelled probes as described^79^. Sequences for the various probes used are as follows: *Axin2*, nucleotides 2077-2877 in Genbank Accession number NM_015732.4; *Foxn1*, nucleotides 2181-3584 in Genbank Accession number XM_006532266.3; *Gcm2*, nucleotides 615-1540 in Genbank Accession number NM_008104.2; *GzmA*, nucleotides 211-1019 in Genbank Accession number M13226.1; *Titf1*, nucleotides 1471-1788 in Genbank Accession number NM_009385.3. Images were acquired on a Zeiss Axioplan 2 microscope equipped with an AxioCam MRc 5 camera.

### Immunofluorescence

Sections from paraformaldehyde (PFA)-fixed, paraffin-embedded tissue were de-paraffinised in xylene, re-hydrated, and then subjected to antigen retrieval with Tris-EDTA buffer (10 mM Tris, 1 mM EDTA, 0.05% Tween 20, pH 9.0) in a steamer for 17 minutes. Sections were subsequently washed in PBS-Tween 20 (0.05%) and permeabilised with 1% TritonX-100. Antibody staining was performed at room temperature in staining buffer (PBS supplemented with 4% serum and 0.05% BSA). Sections were stained for 1 hr with primary antibodies (see Table S1), and then for 30 minutes with secondary antibodies and streptavidin. Sections were washed with PBS-Tween 20 (0.5%) between incubations. After staining sections were mounted in Fluoromount G and images were acquired on a Zeiss Imager Z1 with ApoTome attachment using an Axiocam MRm camera.

### X-gal staining

Cryosections were fixed with fixative solution (0.2% Glutaraldehyde, 0.02% Formaldehyde, 1 mM MgCl_2_ and 0.02% IGEPAL in PBS) for 5 minutes at 4 °C, then washed three times with PBS and covered with X-Gal staining solution (1 mg/mL X-Gal, 5 mM Potassium Ferricyanide, 5 mM Potassium Ferrocyanide, 1 mM MgCl_2_ in PBS) and incubated at 37 °C overnight. Sections were subsequently washed extensively in PBS, and then mounted in Mowiol mounting media for imaging. For whole-mount staining mice were sacrificed, and dissected to ensure adequate penetration of the fixative and staining solutions. After bisection below the diaphragm, the skin covering the cervical region and trunk was removed together with the sternum, and the anterior half of the body was submerged in fixative solution for 30 minutes at 4 °C with agitation, washed through 3 changes of PBS, then submerged in X-Gal staining solution supplemented with 0.01% sodium deoxycholate and incubated at 37 °C overnight with gentle agitation. Stained samples were subsequently rinsed extensively in PBS, then post-fixed with 4% PFA in PBS prior to imaging.

### Flow cytometry

To generate single cell suspensions for TEC staining thymi were finely minced with scissors, and then digested with a cocktail of collagenase type 4 (200 µg/mL), neutral protease (200 µg/mL) and DNaseI (500 ng/mL) in RPMI 1640 + 2% FCS for up to 90 minutes at 37 °C with agitation. Following digestion, EDTA was added to a final concentration of 2 mM to disaggregate any remaining cell clumps. Cells were then washed with RPMI 1640 + 2% FCS, and then re-suspended in PBS/BSA for staining. Thymocyte suspensions for CD4/CD8 staining were generated by gently pressing thymic lobes through 40 µm sieves. Cell surface staining (see Table S1 for antibodies) was performed at 4 °C in PBS supplemented with 0.5% BSA and 0.02% NaN_3_. For detection of β-catenin or E-cadherin, cells were fixed with 2% PFA for 5 minutes on ice, then subsequently washed in permeabilisation buffer (0.5% BSA, 0.1% saponin in PBS), and then stained for intracellular antigens. Antibodies for intracellular staining (see Table S1) were diluted in permeabilisation buffer supplemented with 2% normal rat serum. Although E-cadherin is a cell surface molecule, intracellular staining for E-cadherin expressed by TECs was necessary as the collagenase/dispase treatment required to generate TEC suspensions degrades E-cadherin exposed at the cell surface. E-cadherin trafficking through the endosomes is protected from degradation by collagenase/dispase, and can be detected by intracellular staining.

### Statistical analysis

t-tests (two-tailed) were used to determine the significance levels of the differences between the means of two independent samples, considering equal or unequal variances as determined by the F-test. For multiple tests, the Bonferroni correction was applied.

## Electronic supplementary material

Supplementary Information

## References

[1] Rodewald H-R (2008). Thymus organogenesis. Annu. Rev. Immunol..

[2] Gordon J (2004). Functional evidence for a single endodermal origin for the thymic epithelium. Nat. Immunol..

[3] Gordon J, Bennett AR, Blackburn CC, Manley NR (2001). Gcm2 and Foxn1 mark early parathyroid- and thymus-specific domains in the developing third pharyngeal pouch. Mech. Dev..

[4] Bleul CC, Boehm T (2005). BMP signaling is required for normal thymus development. J. Immunol..

[5] Gordon J, Patel SR, Mishina Y, Manley NR (2010). Evidence for an early role for BMP4 signaling in thymus and parathyroid morphogenesis. Developmental Biology.

[6] Tsai PT, Lee RA, Wu H (2003). BMP4 acts upstream of FGF in modulating thymic stroma and regulating thymopoiesis. Blood.

[7] Bain VE (2016). Tissue-specific roles for sonic hedgehog signaling in establishing thymus and parathyroid organ fate. Development.

[8] Balciunaite G (2002). Wnt glycoproteins regulate the expression of FoxN1, the gene defective in nude mice. Nat. Immunol..

[9] Manley NR, Capecchi MR (1998). Hox group 3 paralogs regulate the development and migration of the thymus, thyroid, and parathyroid glands. Developmental Biology.

[10] Chojnowski JL (2014). Multiple roles for HOXA3 in regulating thymus and parathyroid differentiation and morphogenesis in mouse. Development.

[11] Su DM, Manley NR (2000). Hoxa3 and pax1 transcription factors regulate the ability of fetal thymic epithelial cells to promote thymocyte development. J. Immunol..

[12] Peters H, Neubüser A, Kratochwil K, Balling R (1998). Pax9-deficient mice lack pharyngeal pouch derivatives and teeth and exhibit craniofacial and limb abnormalities. Genes Dev..

[13] Hetzer-Egger C (2002). Thymopoiesis requires Pax9 function in thymic epithelial cells. Eur. J. Immunol..

[14] Laclef C, Souil E, Demignon J, Maire P (2003). Thymus, kidney and craniofacial abnormalities in Six 1 deficient mice. Mech. Dev..

[15] Xu P-X (2002). Eya1 is required for the morphogenesis of mammalian thymus, parathyroid and thyroid. Development.

[16] Zou D (2006). Patterning of the third pharyngeal pouch into thymus/parathyroid by Six and Eya1. Developmental Biology.

[17] Jerome LA, Papaioannou VE (2001). DiGeorge syndrome phenotype in mice mutant for the T-box gene, Tbx1. Nat. Genet..

[18] Revest JM, Suniara RK, Kerr K, Owen JJ, Dickson C (2001). Development of the thymus requires signaling through the fibroblast growth factor receptor R2-IIIb. J. Immunol..

[19] Gardiner JR (2012). Localised inhibition of FGF signalling in the third pharyngeal pouch is required for normal thymus and parathyroid organogenesis. Development.

[20] Foster KE (2010). EphB-ephrin-B2 interactions are required for thymus migration during organogenesis. Proc. Natl. Acad. Sci. USA.

[21] Mendelsohn C (1994). Function of the retinoic acid receptors (RARs) during development (II). Multiple abnormalities at various stages of organogenesis in RAR double mutants. Development.

[22] Mulder GB, Manley N, Maggio-Price L (1998). Retinoic acid-induced thymic abnormalities in the mouse are associated with altered pharyngeal morphology, thymocyte maturation defects, and altered expression of Hoxa3 and Pax1. Teratology.

[23] Niederreither K (2003). The regional pattern of retinoic acid synthesis by RALDH2 is essential for the development of posterior pharyngeal arches and the enteric nervous system. Development.

[24] Frank DU (2002). An Fgf8 mouse mutant phenocopies human 22q11 deletion syndrome. Development.

[25] Abu-Issa R, Smyth G, Smoak I, Yamamura K-I, Meyers EN (2002). Fgf8 is required for pharyngeal arch and cardiovascular development in the mouse. Development.

[26] Schneider MR, Kolligs FT (2015). E-cadherin’s role in development, tissue homeostasis and disease: Insights from mouse models: Tissue-specific inactivation of the adhesion protein E-cadherin in mice reveals its functions in health and disease. Bioessays.

[27] Lee MG, Sharrow SO, Farr AG, Singer A, Udey MC (1994). Expression of the homotypic adhesion molecule E-cadherin by immature murine thymocytes and thymic epithelial cells. J. Immunol..

[28] Müller KM, Luedecker CJ, Udey MC, Farr AG (1997). Involvement of E-cadherin in thymus organogenesis and thymocyte maturation. Immunity.

[29] Zuklys S (2009). Stabilized beta-catenin in thymic epithelial cells blocks thymus development and function. The Journal of Immunology.

[30] Brembeck FH, Rosário M, Birchmeier W (2006). Balancing cell adhesion and Wnt signaling, the key role of beta-catenin. Curr. Opin. Genet. Dev..

[31] Logan CY, Nusse R (2004). The Wnt signaling pathway in development and disease. Annu. Rev. Cell Dev. Biol..

[32] Grigoryan T, Wend P, Klaus A, Birchmeier W (2008). Deciphering the function of canonical Wnt signals in development and disease: conditional loss- and gain-of-function mutations of beta-catenin in mice. Genes Dev..

[33] Mulroy T, McMahon JA, Burakoff SJ, McMahon AP, Sen J (2002). Wnt-1 and Wnt-4 regulate thymic cellularity. Eur. J. Immunol..

[34] Kuraguchi M (2006). Adenomatous polyposis coli (APC) is required for normal development of skin and thymus. PLoS Genet..

[35] Kvell K (2010). Wnt4 and LAP2alpha as pacemakers of thymic epithelial senescence. PLoS ONE.

[36] Varecza Z (2011). Multiple suppression pathways of canonical Wnt signalling control thymic epithelial senescence. Mech. Ageing Dev..

[37] Heinonen KM (2011). Wnt4 regulates thymic cellularity through the expansion of thymic epithelial cells and early thymic progenitors. Blood.

[38] Liang C-C (2013). Thymic epithelial β-catenin is required for adult thymic homeostasis and function. Immunol. Cell Biol..

[39] Brunk F, Augustin I, Meister M, Boutros M, Kyewski B (2015). Thymic Epithelial Cells Are a Nonredundant Source of Wnt Ligands for Thymus Development. The Journal of Immunology.

[40] Kvell K, Fejes AV, Parnell SM, Pongracz JE (2014). Active Wnt/beta-catenin signaling is required for embryonic thymic epithelial development and functionality *ex vivo*. Immunobiology.

[41] Pongrácz J, Hare K, Harman B, Anderson G, Jenkinson EJ (2003). Thymic epithelial cells provide WNT signals to developing thymocytes. Eur. J. Immunol..

[42] Maretto S (2003). Mapping Wnt/beta-catenin signaling during mouse development and in colorectal tumors. Proc. Natl. Acad. Sci. USA.

[43] Ishitani T (1999). The TAK1-NLK-MAPK-related pathway antagonizes signalling between beta-catenin and transcription factor TCF. Nature.

[44] Ota S (2012). NLK positively regulates Wnt/β-catenin signalling by phosphorylating LEF1 in neural progenitor cells. EMBO J.

[45] Ishitani T, Ishitani S (2013). Nemo-like kinase, a multifaceted cell signaling regulator. Cell. Signal.

[46] Nehls M, Pfeifer D, Schorpp M, Hedrich H, Boehm T (1994). New member of the winged-helix protein family disrupted in mouse and rat nude mutations. Nature.

[47] Nehls M (1996). Two genetically separable steps in the differentiation of thymic epithelium. Science.

[48] Lee, D., Prowse, D. M. & Brissette, J. L. Association between MousenudeGene Expression and the Initiation of Epithelial Terminal Differentiation. **208**, 362–374 (1999). 10.1006/dbio.1999.9221 10191051

[49] Ebnet K, Levelt CN, Tran TT, Eichmann K, Simon MM (1995). Transcription of granzyme A and B genes is differentially regulated during lymphoid ontogeny. J. Exp. Med..

[50] Wong MH, Rubinfeld B, Gordon JI (1998). Effects of forced expression of an NH2-terminal truncated beta-Catenin on mouse intestinal epithelial homeostasis. J. Cell Biol..

[51] Niehrs C (2012). The complex world of WNTreceptor signalling. Nat. Rev. Mol. Cell Biol..

[52] Kutlesa S (2002). E-cadherin-mediated interactions of thymic epithelial cells with CD103+ thymocytes lead to enhanced thymocyte cell proliferation. J. Cell. Sci..

[53] Tinkle CL, Lechler T, Pasolli HA, Fuchs E (2004). Conditional targeting of E-cadherin in skin: insights into hyperproliferative and degenerative responses. Proc. Natl. Acad. Sci. USA.

[54] Posthaus H (2002). beta-Catenin is not required for proliferation and differentiation of epidermal mouse keratinocytes. J. Cell. Sci..

[55] Ishida T (2013). Ectopic cervical thymus associated with infant death: 2 case reports and literature review. International Journal of Pediatric Otorhinolaryngology.

[56] Rahbar R (2008). Lingual thyroid in children: a rare clinical entity. Laryngoscope.

[57] Terszowski G (2006). Evidence for a Functional Second Thymus in Mice. Science.

[58] Dooley J, Erickson M, Gillard GO, Farr AG (2006). Cervical thymus in the mouse. J. Immunol..

[59] Panigraphi D, Waxler GL, Mallman VH (1971). The thymus in the chicken and its anatomical relationship to the thyroid. J. Immunol..

[60] Jordan RK (1976). Development of sheep thymus in relation to in utero thymectomy experiments. Eur. J. Immunol..

[61] Symington J (1898). The Thymus Gland in the Marsupialia. J Anat Physiol.

[62] Kim DW (2013). The prevalence and features of thyroid pyramidal lobe, accessory thyroid, and ectopic thyroid as assessed by computed tomography: a multicenter study. Thyroid.

[63] Prakash (2012). Variations in the anatomy of the thyroid gland: clinical implications of a cadaver study. Anat Sci Int.

[64] Takanashi Y (2015). Pyramidal lobe of the thyroid gland and the thyroglossal duct remnant: a study using human fetal sections. Ann. Anat..

[65] Hickman, W. *Congenital tumor of the base of the tongue, pressing down the epiglottis on the larynx and causing death by suffocation sixteen hours after birth*. (Trans Pathol Soc Lond, 1869).

[66] Baldwin RL, Copeland SK (1988). Lingual thyroid and associated epiglottitis. South. Med. J..

[67] Ohbuchi T (2012). Coexistence of pyriform sinus fistula, ectopic lingual thyroid, and ectopic cervical thymus. Auris Nasus Larynx.

[68] Bubuteishvili L, Garel C, Czernichow P, Léger J (2003). Thyroid abnormalities by ultrasonography in neonates with congenital hypothyroidism. J. Pediatr..

[69] McLean G (2012). Solid ectopic cervical thymus in neonates with thyroid agenesis. J Ultrasound Med.

[70] Louis I (2008). The signaling protein Wnt4 enhances thymopoiesis and expands multipotent hematopoietic progenitors through beta-catenin-independent signaling. Immunity.

[71] Heinonen KM, Vanegas JR, Lew D, Krosl J, Perreault C (2011). Wnt4 enhances murine hematopoietic progenitor cell expansion through a planar cell polarity-like pathway. PLoS ONE.

[72] Van-Amerongen R (2012). Alternative Wnt pathways and receptors. Cold Spring Harb Perspect Biol.

[73] Huh SH, Ornitz DM (2010). Beta-catenin deficiency causes DiGeorge syndrome-like phenotypes through regulation of Tbx1. Development.

[74] Kameda Y, Ito M, Nishimaki T, Gotoh N (2009). FRS2alpha is required for the separation, migration, and survival of pharyngeal-endoderm derived organs including thyroid, ultimobranchial body, parathyroid, and thymus. Dev. Dyn..

[75] Kortenjann M (2001). Abnormal bone marrow stroma in mice deficient for nemo-like kinase, Nlk. Eur. J. Immunol..

[76] Brault V (2001). Inactivation of the beta-catenin gene by Wnt1-Cre-mediated deletion results in dramatic brain malformation and failure of craniofacial development. Development.

[77] Boussadia O, Kutsch S, Hierholzer A, Delmas V, Kemler R (2002). E-cadherin is a survival factor for the lactating mouse mammary gland. Mech. Dev..

[78] Soza-Ried C, Bleul CC, Schorpp M, Boehm T (2008). Maintenance of Thymic Epithelial Phenotype Requires Extrinsic Signals in Mouse and Zebrafish. J. Immunol..

[79] Breitschopf H, Suchanek G, Gould RM, Colman DR, Lassmann H (1992). *In situ* hybridization with digoxigenin-labeled probes: sensitive and reliable detection method applied to myelinating rat brain. Acta Neuropathol..

