# Supplementary material for: Elevated levels of Wnt signaling disrupt thymus morphogenesis and function
Source: Sci Rep. 2017 Apr 11;7:785. doi: 10.1038/s41598-017-00842-0 (PMC5429746; doi:10.1038/s41598-017-00842-0)
Supplement: Supplementary file 1 — Supplementary Information [file 41598_2017_842_MOESM1_ESM.pdf]

Supplementary information for

Elevated levels of Wnt signaling disrupt thymus morphogenesis and function

Jeremy B. Swann, Christiane Happe and Thomas Boehm

Figure S1.

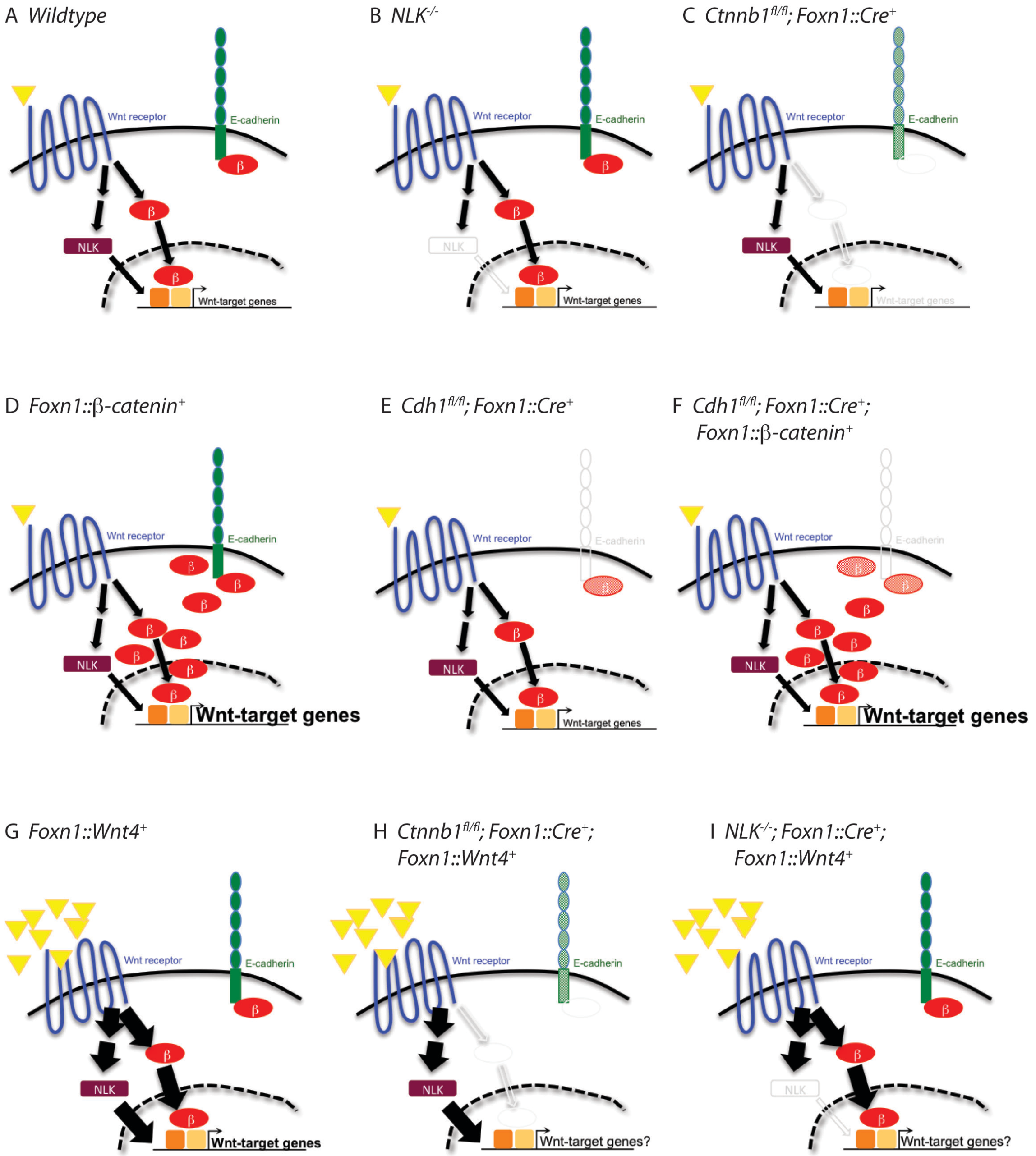

Supplementary Figure 1. Schematic depiction of the various genetic models used in the study. (A) A wild-type TEC is depicted, where binding of Wnts (yellow triangle) to a Wnt-receptor expressed at the surface initiates β-catenin-dependent signaling by stabilisation of β-catenin (red ovals), which translocates to the nucleus, where it modulates expression of target genes via interactions with transcriptional co-activators (orange boxes). Additionally, Wnt binding can activate signaling via NLK (purple rectangle), which can modulate transcriptional output by phosphorylating downstream effectors. E-cadherin is present at the TEC surface, and interacts with β-catenin via its intracellular tail to maintain intracellular adhesive interactions. (B) Depicts the loss of

NLK-dependent signaling in *Nlk*-deficient mice. (C) Loss of β-catenin-dependent signaling in β-catenin-deficient mice is shown; note that in this situation, although E-cadherin is still expressed, adhesive functions are compromised. (D) Depicts the overexpression of stabilised β-catenin, resulting in enhanced expression of Wnt-target genes, and potential modification of E-cadherin-mediated intracellular adhesion. (E) E-cadherin-deficiency is depicted; in this situation the fate of the β-catenin that would normally be associated with E-cadherin at the cell surface is unclear, although it is likely degraded by the proteasome. (F) The combined loss of E-cadherin and overexpression of stabilised β-catenin is depicted. (G) Indicates the enhanced Wnt-signaling induced by overexpression of Wnt4. (H - I) Depict the overexpression of Wnt4 in the absence of β-catenin or NLK respectively. Note that in (G-H), in addition to its effect on TECs, the overexpressed Wnt4 can potentially exert paracrine effects on other cells, such as those forming the parathyroid and the thyroid.

Figure S2.

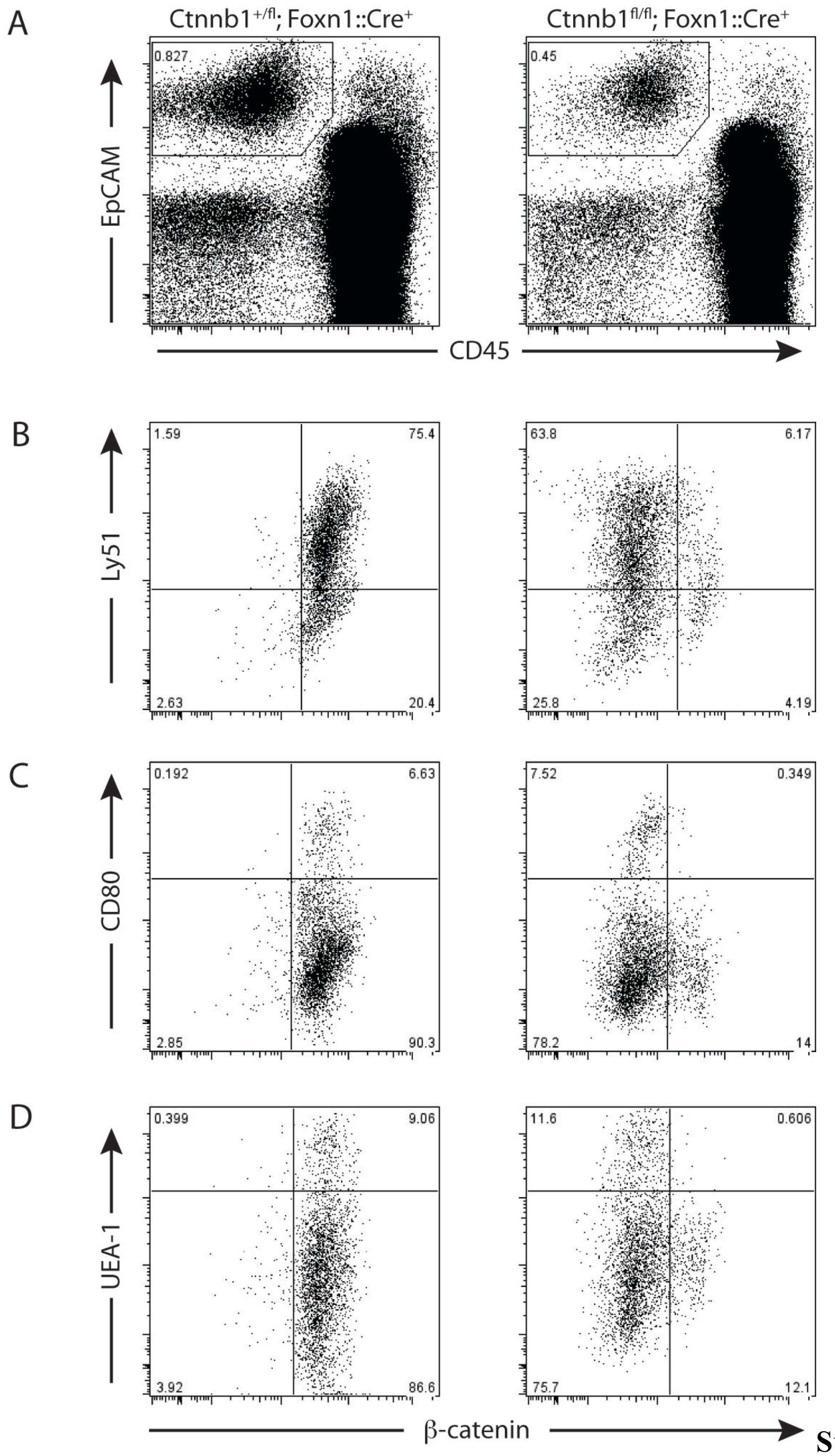

Supplementary

Figure 2. Flow cytometric analysis of TEC subsets in newborn β-catenin-deficient thyme. (A) Representative gating on $CD45^{-}EpCAM^{+}$ TECs is shown.
(B - D) Staining for Ly51 (B), CD80 (C) or UEA-1 (D) in combination with intracellular β-catenin staining is depicted. Plots B - D are gated on TECs.

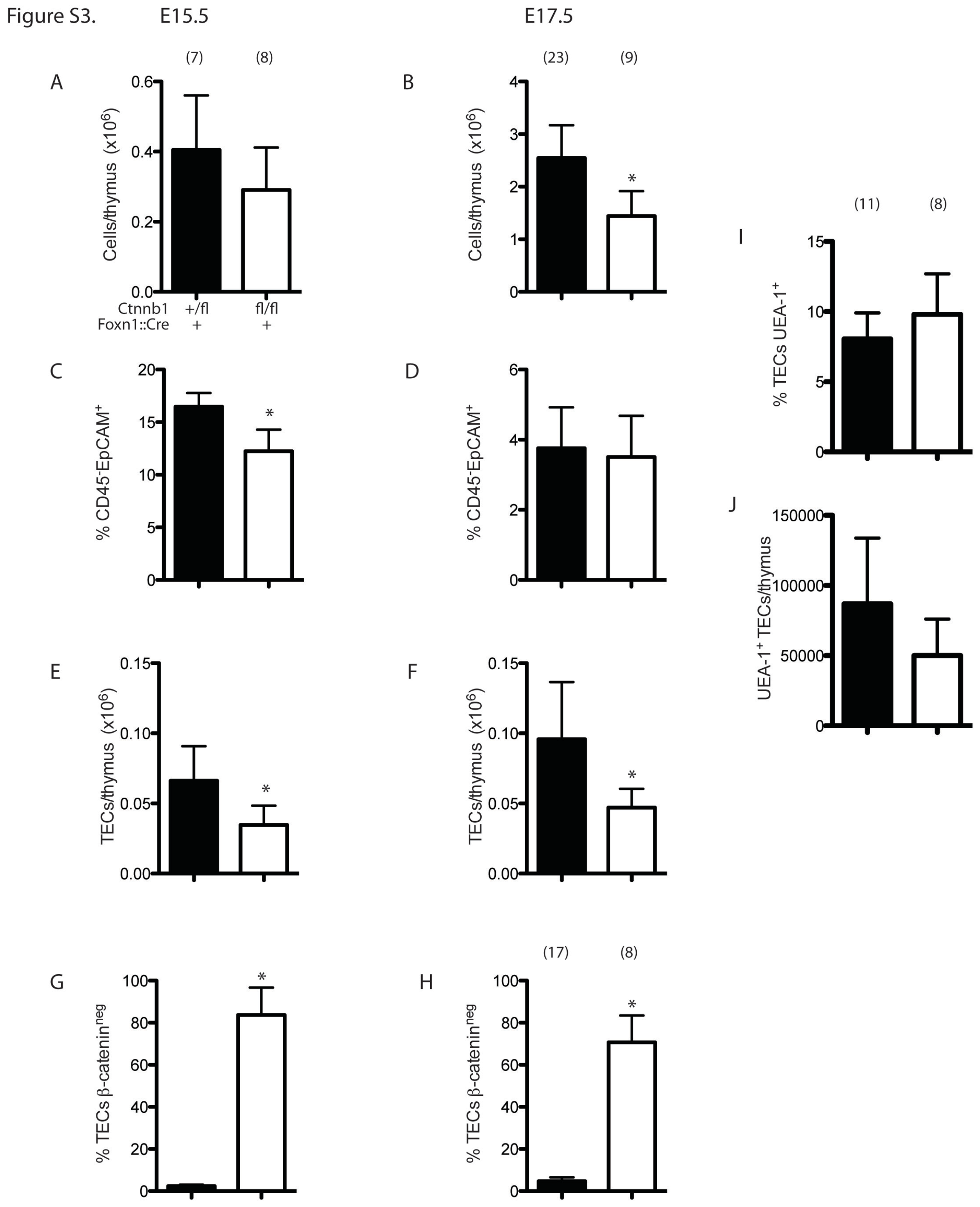

Supplementary Figure 3. Flow cytometric analysis of embryonic, β-catenin-deficient thymi. Thymi were isolated from E15.5 (A, C, E & G) or E17.5 (B, D, F, H - J) embryos and analysed for total cellularity (A & B), TEC proportions (C & D), TEC number (E &

F) and β-catenin deletion efficiency (G & H). The proportion (I) and number (J) of UEA-1^+ TECs was also analysed at E17.5.

Figure S4.

A

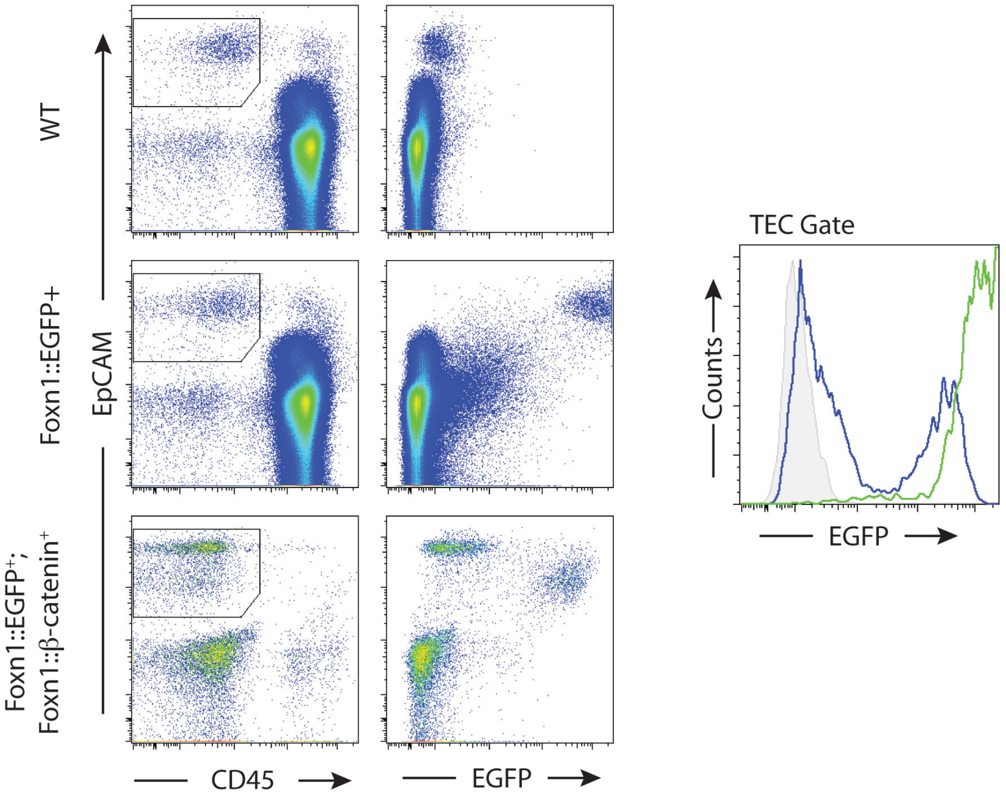
WT
Foxn1::EGFP+
Foxn1::EGFP+;
Foxn1::β-catenin+
EpCAM
CD45
EGFP
TEC Gate
Counts
EGFP

B

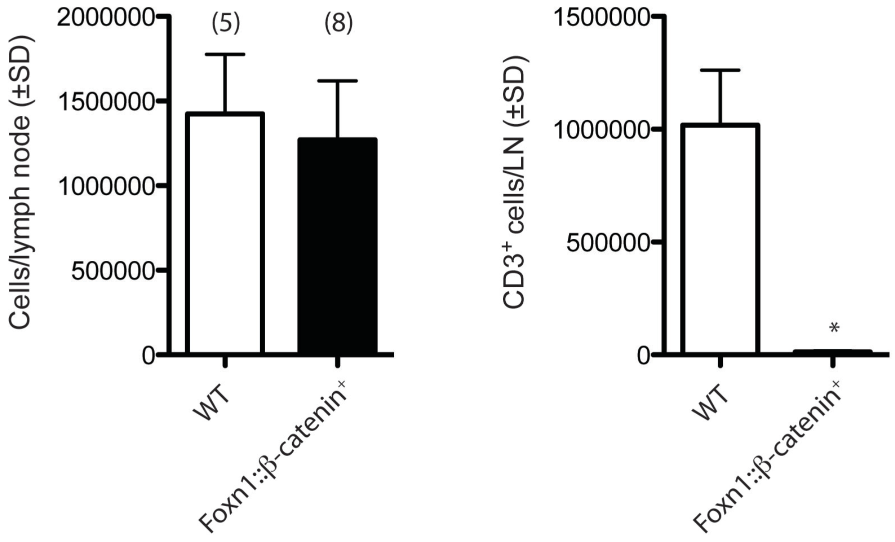
2000000
1500000
1000000
500000
0
(5)
(8)
Cells/lymph node (±SD)
WT
Foxn1::β-catenin+
1500000
1000000
500000
0
CD3+ cells/LN (±SD)
*
WT
Foxn1::β-catenin+

Supplementary Figure S4. Flow cytometric analysis of β-catenin-transgenic mice. (A) Thymi were harvested from three-day old wild-type (WT), *Foxn1::EGFP* or *Foxn1::EGFP;Foxn1::β-catenin*-transgenic mice and analysed by flow cytometry. *Foxn1::EGFP;Foxn1::β-catenin*-double transgenic lobes were collected by dissecting the cervical region under UV-illumination to identify EGFP-expressing tissue. After isolation, thymic tissue was digested with collagenase/dispase to generate a single cell suspension, and stained with CD45 and EpCAM to identify TECs. Representative TEC gates are depicted in the left column; EGFP expression relative to EpCAM is depicted in the right column. EGFP expression by gated TEC populations is depicted as a histogram overlay; the blue tracing represents EGFP expression by *Foxn1::EGFP; Foxn1::β-catenin* double-transgenic TECs, compared to TECs from *Foxn1::EGFP* single-transgenic (in green), or WT mice (grey). It should be noted that due to the failed separation of the thymus and parathyroid in β-catenin-transgenic mice, some of the $CD45^{-}EpCAM^{+}$ epithelial cells could potentially be derived from thymus-associated parathyroid tissue. Nevertheless, EGFP expression is reduced in double-transgenic mice compared to *Foxn1::EGFP* single-transgenic mice, indicating a reduction in *Foxn1* promoter activity. (B) Total cell counts, and numbers of $CD3^{+}$ cells in the peripheral lymph nodes of adult WT and *β-catenin*-transgenic mice are depicted as mean ± SD.

Figure S5.

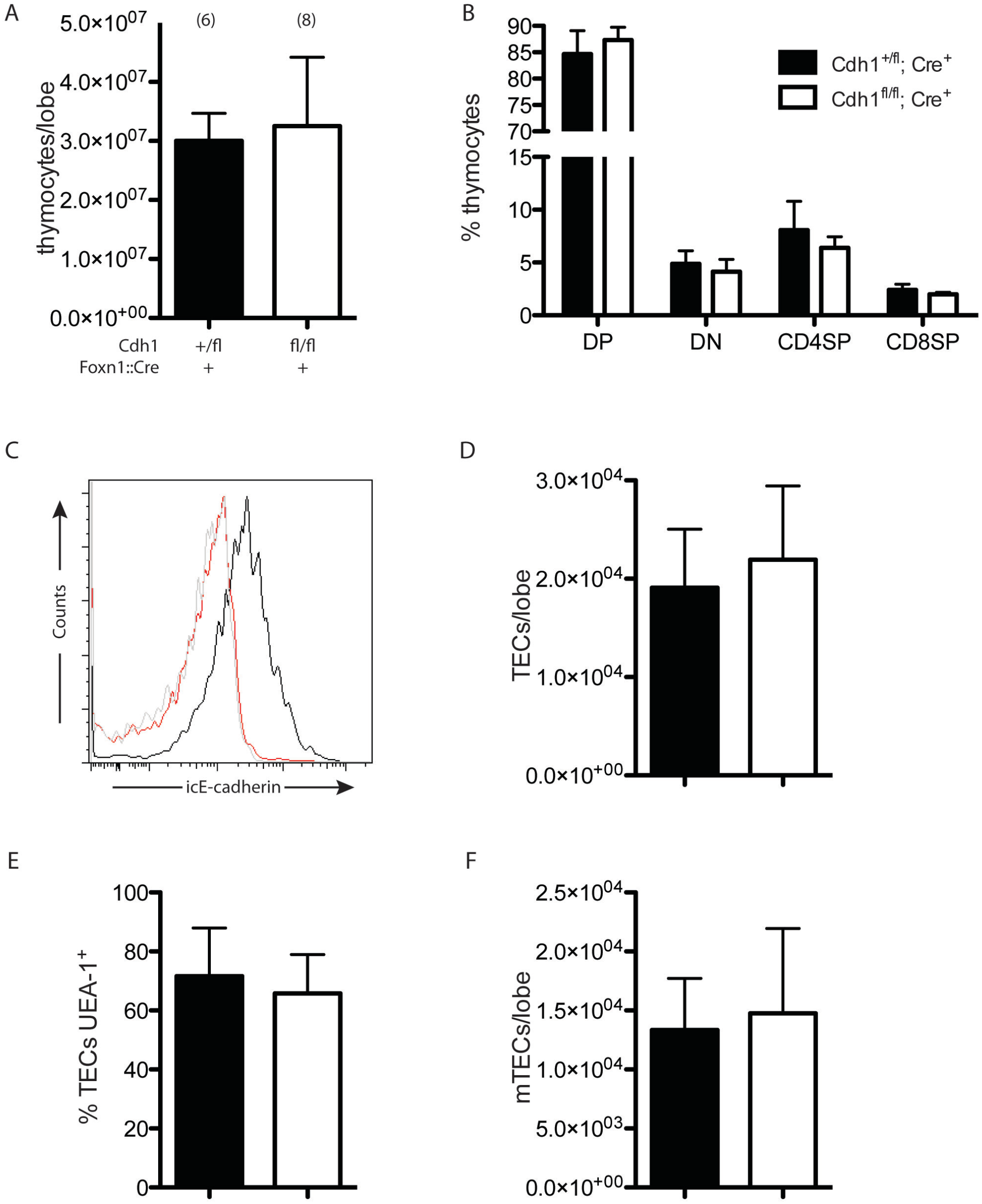

Supplementary Figure 5. Flow cytometric analysis of adult E-cadherin-deficient mice. (A) Thymocyte counts from 12-week old control and E-cadherin-deficient mice. (B) Proportions of thymocytes subsets at 12 weeks. (C) Intracellular staining for E-cadherin expressed by gated $CD45^{-}EpCAM^{+}$ TECs derived from control (black tracing) or E-cadherin-deficient mice (red tracing), compared to isotype control staining (in grey). (D) Total TEC numbers. (E) Proportions of UEA-1^{+} TECs. (F) Numbers of UEA-1^{+} mTECs are shown. All column graphs depict the mean ± SD for control (black bars,

Cdh1$^{+/fl}$; Foxn1::Cre^{+}) and E-cadherin-deficient (open bars, Cdh1$^{fl/fl}$; Foxn1::Cre^{+}) mice aged 12 weeks.

Figure S6.

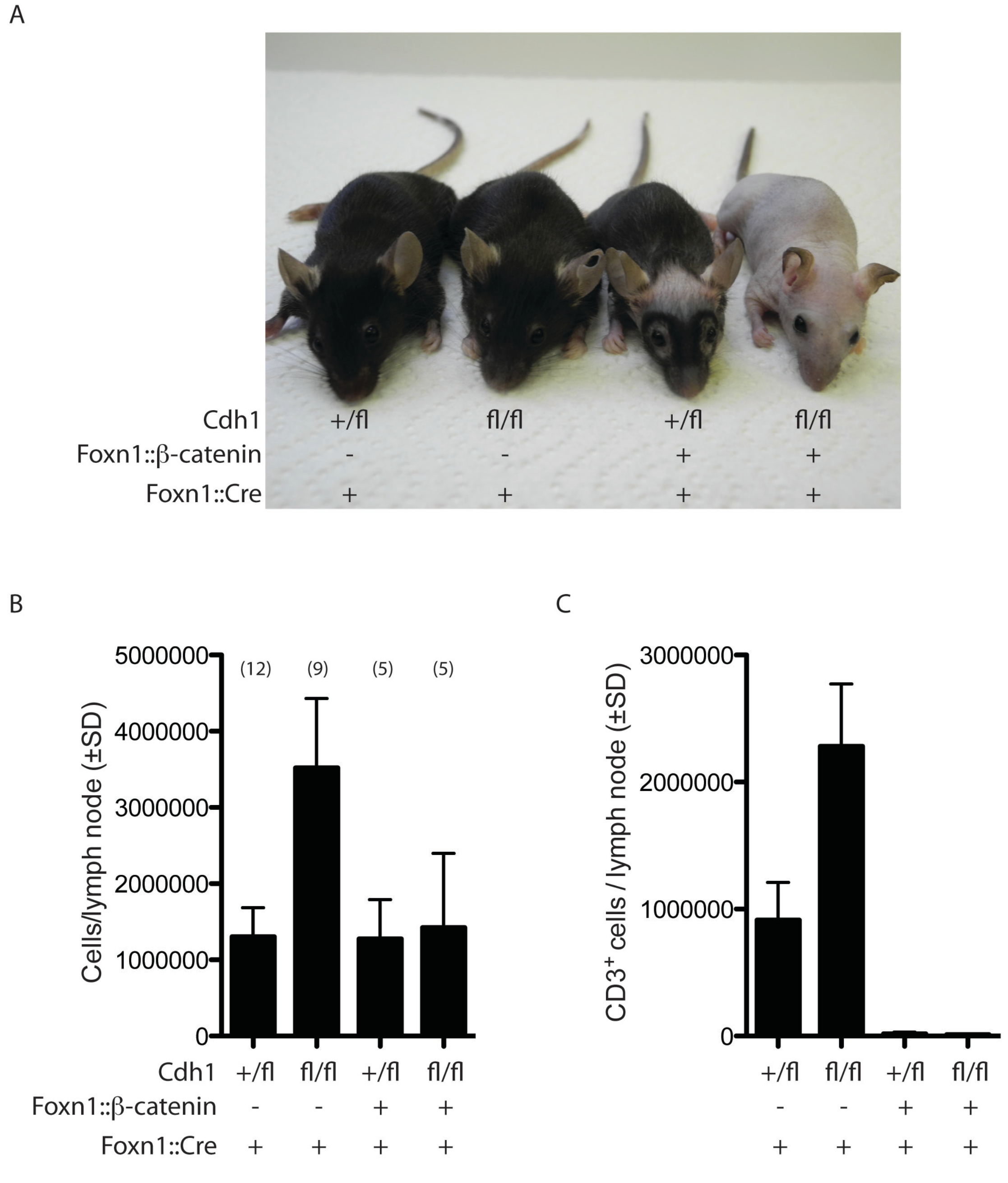

Supplementary Figure 6. E-cadherin-deficiency fails to rescue T cell development in Foxn1::β-catenin-transgenic mice. (A) Representative coat phenotypes are shown for the indicated genotypes. (B) The number of total cells/lymph node, and (C) Numbers of $CD3^+$ T cells/lymph node is shown for each genotype. Data was collected from 12-week old mice, and is presented as mean ± SD.

Figure S7.

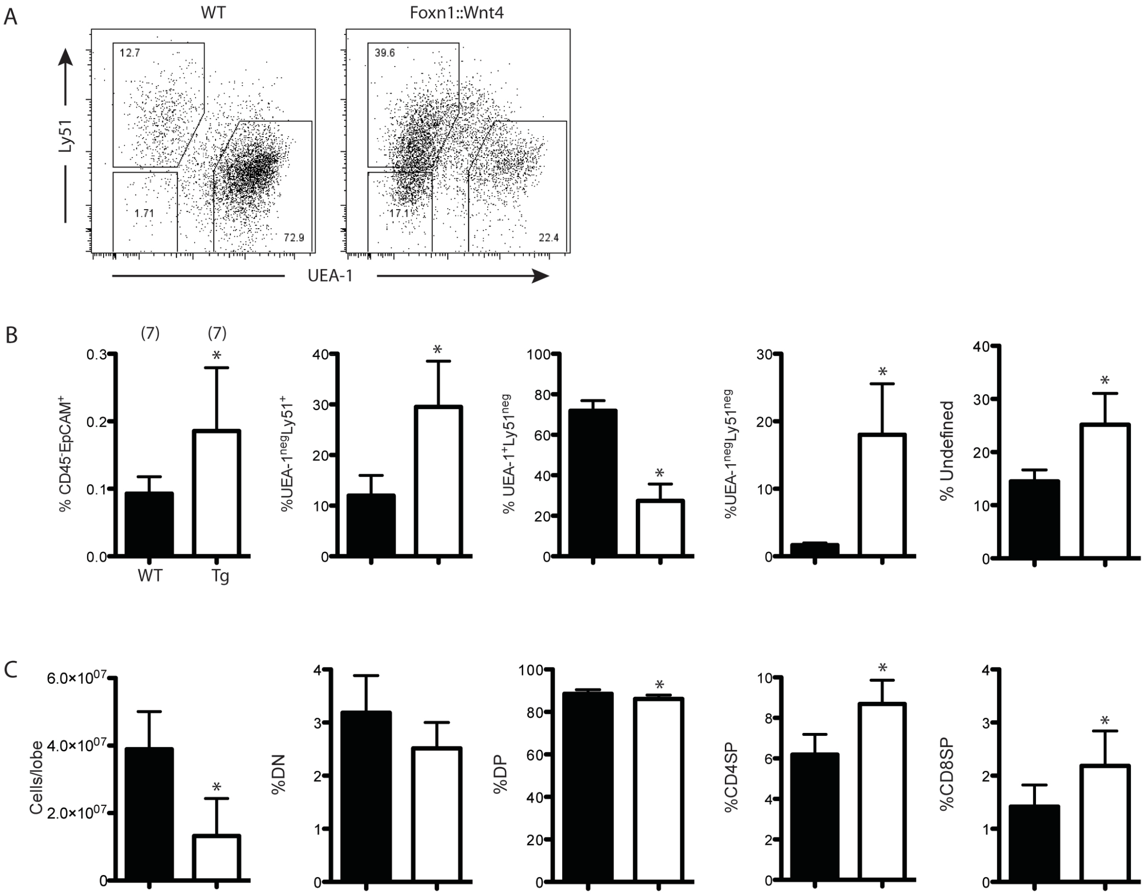

Supplementary Figure 7. Thymus function in adult Foxn1::Wnt4-transgenic mice. (A - D) The TEC (A&B), thymoycte (C) and peripheral T cell subsets (D) of 12-week old WT (black bars) and *Foxn1::Wnt4*$^{+}$ mice (open bars) were analysed by flow cytometry. (A) The gating strategy for defining TEC subsets is shown in (A), which depicts UEA-1 and Ly51 staining of gated CD45^{-}EpCAM^{+} TECs. (B) Proportions of total TECs, together with the various TEC subsets as defined in (A). (C) The total number of thymocytes, and proportions of the various thymocyte subsets are shown.

Supplementary Table 1. Antibodies and staining reagents for flow cytometry and immunofluorescence

Antigen/Reagent	Clone	Conjugate	Supplier
β-catenin	14/beta-catenin	-	BD Biosciences
CD3	145-2C11	APC	eBioscience
CD4	GK1.5	FITC	BioLegend
CD8	53-6.7	PE	eBioscience
CD19	1D3	PE Cy7	eBioscience
CD44	1M7	PE	BD Bioscience
CD45	30-F11	PE Cy7	BioLegend
CD62L	MEL-14	FITC	eBioscience
CD80	16-10A1	Biotin	BioLegend
E-cadherin	Decma-1	none, Alexa Fluor 647	eBioscience
EpCAM	G8.8	APC	BioLegend
Keratin 5	rabbit polyclonal	-	Covance
Keratin 8	Troma-1	-	in house
Ly51	6C3	PE	eBioscience
MHC2	M5/114.15.2	FITC	BioLegend
mouse IgG1	goat polyclonal	Biotin	SouthernBiotech
mouse IgG (H+L)	rat polyclonal	FITC	Jackson ImmunoResearch
rabbit IgG (H+L)	goat polyclonal	Alexa Fluor 488	Invitrogen
rat IgG (H+L)	donkey polyclonal	Cy3	Jackson ImmunoResearch
Streptavidin	-	Cy3, Cy5	Jackson ImmunoResearch
Streptavidin	-	FITC, PE or Alexa Fluor v450	eBioscience
UEA-1	-	Biotin, FITC	Vector Laboratories
